# A new mesoporous Ce–Mn-LDH-based Co-MOF nano-composite for the green synthesis of tetrazoloquinazolines†

**DOI:** 10.1039/d4na00643g

**Published:** 2024-11-21

**Authors:** Samira Javadi, Davood Habibi

**Affiliations:** a Department of Organic Chemistry, Faculty of Chemistry and Petroleum Sciences, Bu-Ali Sina University Hamedan Iran davood.habibi@gmail.com dhabibi@basu.ac.ir +98-81-31408025 +98-81-38380922

## Abstract

Tannic acid (TA), as a plant polyphenol, has many active sites for chelation with metals, so TA-oligomers (TA-Olig) were used for the first time as ligands on the surface of Ce–Mn-LDH to prepare the layered double hydroxide-based metal–organic framework (Ce–Mn-LDH@CPTMS@TA-Olig@Co-MOF = E) nanocomposite. In this regard, a homogeneous water/ethanol solution was prepared by sol–gel method using polyethylene glycol and ammonia solution, and then TA was converted into a set of oligomers in the presence of formaldehyde. In the next step, Ce–Mn-LDH was prepared in a ratio of 1 : 4 of Ce to Mn, modified with 3-chloropropylmethoxysilane, functionalized by TA-Olig, and then cobalt salt was used to prepare E. Finally, the structure of E was determined by FT-IR, ICP, XRD, BET, EDX, SEM, SEM-mapping, TEM, and TGA-DTA techniques and used as a new and potent nanocatalyst for the synthesis of tetrazoloquinazolines a(1–12). One of the advantages of this nanocatalyst is the active surface to form products in a limited time in mild conditions with high efficiency and easy separation. Also, the catalytic activity is maintained even after four consecutive runs.

## Abstract 2

Tannic acid (TA), as a plant polyphenol, has active sites for chelation, so TA-Olig were used as ligands on the surface of Ce–Mn-LDH to prepare the layered double hydroxide-based metal–organic framework (Ce–Mn-LDH@CPTMS@TA-Olig@Co-MOF = E).

## Graphical abstract

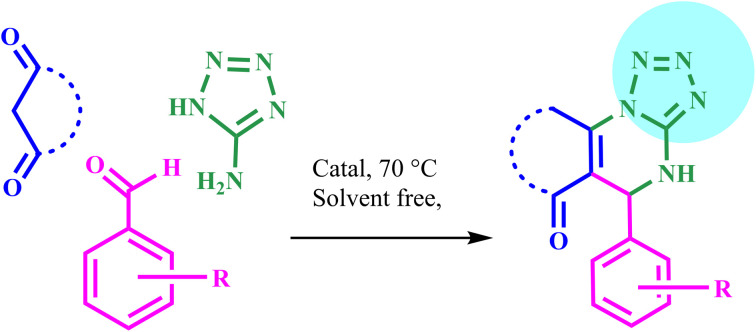

## 1. Introduction

The spherical structure of colloidal particles, with diameters ranging from 0.01 to 1 μm, has been extensively studied in fields such as catalysis, drug delivery, medicine, material science, and biology.^1^

Among the various approaches for preparing stable colloidal particles, the Stöber method has been developed. A key strategy in this method is the preparation of silica-containing colloidal particles using the sol–gel process, which creates a homogeneous system for colloidal particles with a uniform structure, facilitating the synthesis of various types of polymer and mineral spheres, such as resorcinol-formaldehyde spheres.^2,3^ Coordination polymers (CPs) or metal–organic frameworks (MOFs) are networks composed of organic linkers and metal centers. They exhibit diverse structures and tunable particle sizes, which have attracted significant attention over the last two decades.^4,5^

Metal-phenol coordination polymers (MPCPs) are a subset of metal–organic coordination polymers that are typically prepared using plant polyphenols, such as TA, as organic ligands. The interaction between metal species and catechol groups facilitates their formation. Owing to their high surface area, MPCPs find applications in biomedicine, sensors, and catalysis.^6,7^ TA is a natural polyphenol from the phenolic acid group, consisting of a central glucose unit with ten gallic acid molecules attached to it.^8,9^ A unique feature of TA is the strong interaction between catechol groups and metal ions, which leads to extensive chemical compatibility, especially in metal ion complexes.^10^

The design of nanoporous solid materials from two-dimensional materials, such as transition metal dichalcogenides (TMDs), layered double hydroxides (LDHs), graphene oxide, *etc.*, has attracted much attention nowadays.^11–13^ LDHs, or pseudo hydrotalcite, are a group of two-dimensional layered solids in which divalent and trivalent metal cations, as well as interlayer anions, are present in their semi-hydrophilic structure.^14^

The integration of two MOF and LDH structures into a composite with unique properties is a smart design to increase the catalytic performance.^15^ In this method, the uniform growth of MOF crystals on LDH plates results in the formation of organic-mineral hybrid materials with high porosity and surface area, which are used in the fields of catalysis, energy storage, absorption, and optoelectronics.^16,17^ Therefore, synthesizing these composites *via* a simple, cost-effective, and environmentally friendly method is of great importance.^18^ In most studies, these composites have been used as absorbers for the removal of metal pollutants from the environment due to their high porosity. In this work, we have utilized them as a green and powerful catalyst for the synthesis of quinazolines. For example, Soltani and colleagues, in 2021, utilized MOF growth on NiCo-LDH to synthesize a NiCo-LDH/MOF nanocomposite for removing metal ions from an aqueous medium.^19^ Mallakpour and colleagues modified the surface of LDH with chitosan and TA to absorb reactive dyes.^20^

Quinazolines are nitrogen-containing heterocycles that were prepared by Gabriel in 1903, with other names such as phenazine, 6-benzopyrimidine, and benzo-1,3-diazine also known. Quinazolines are nitrogen-containing cyclic heterocycles that were prepared by Gabriel in 1903, with other names such as phenazine, 6-benzopyrimidine, and benzo-1,3-diazine also known.^21^ Quinazolines and tetrazole-pyrimidines indeed have a wide field of biological activities,^22,23^ and are known for their anti-cancer, anti-diabetic, anti-inflammatory, anti-microbial, anti-hypertensive, and anti-convulsant.^24,25^ Therefore, the synthesis of tetrazole-pyrimidines was reported by Ba_0.5_Sr_0.5_Fe_12_O_19_@PU-SO_3_H,^26^*p*-TsOH,^27^ Fe_3_O_4_@C/Ph-SO_3_H,^28^ TsOH,^29^ AlCl_3_,^30^ and Fe_3_O_4_@PEG-400-SO_3_H.^31^

**Scheme 1:**
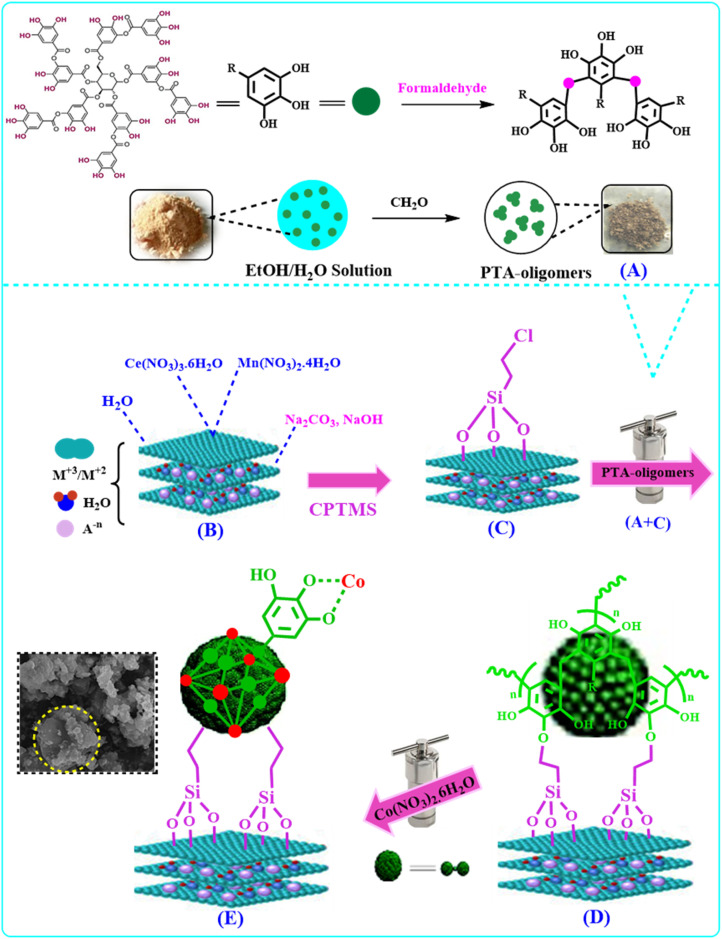
Synthesis of E.

In this work, based on the principles of green chemistry, TA was used to prepare corresponding TA-Olig with abundant catechol groups to be chelated to metal to form a coordinated metal-phenolic compound in an easy and green way. So, Ce–Mn-LDH was used as a scaffold for the preparation of the stable metal-phenolic coordinated compound (Scheme 1).

**Scheme 2:**
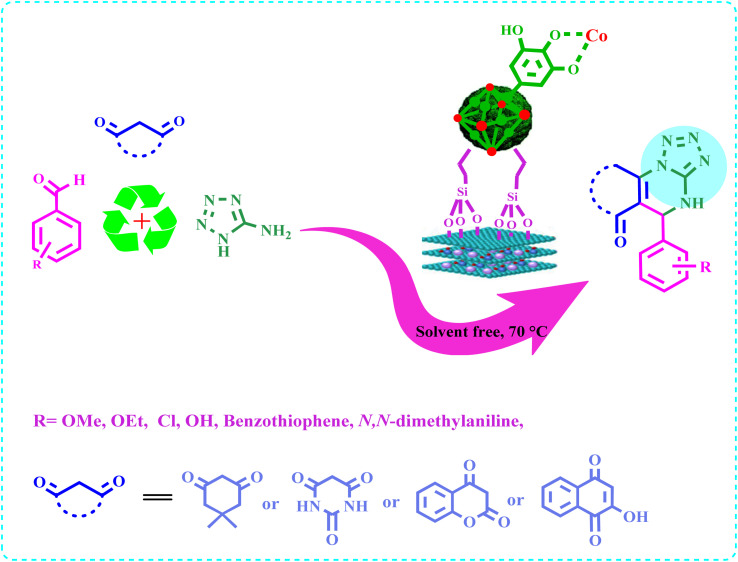
Synthesis of a(1–12).

Finally, E was characterized by FT-IR, ICP, XRD, EDX, SEM, SEM-mapping, TEM, and TGA-DTA techniques and used as a potent nanocatalyst for the green synthesis of tetrazolo-[1,5-*a*]quinazolines a(1–12) from the three component condensation reaction of 5-amino-tetrazole, aldehydes, and diketones under solvent-free conditions at 70 °C with short reaction times and high yields (Scheme 2).

## 2. Experimental

### 2.1. Material

All chemicals [Ce(NO_3_)_3_·6H_2_O, Mn(NO_3_)_2_·4H_2_O, Co(NO_3_)_2_·6H_2_O, TA, Na_2_CO_3_, NaOH, toluene, and ethanol] were purchased from the Aldrich and Merck chemical companies and used as received.

### 2.2. Equipment

Melting points were determined by A BUCHI 510 device in open capillary tubes. FT-IR spectra were recorded on a PerkinElmer GX spectrophotometer using KBr. ^1^H and ^13^C NMR spectra were recorded on a BRUKER AVANCE 300 MHz instrument in DMSO-*d*_6_. Crystalline structures of products were identified on a Bruker D_8_ Advance XRD instrument with Cu Kα as the incident radiation (*λ* = 1/56 054 Å) (40 kV and 100 mA). The catalyst particles' morphology was determined *via* the SEM images on a Philips XL-30 operated at 30 kV accelerating voltage. Qualitative detection of the Ce and Mn elements was performed by EDX in an ESEM (SIGM, Germany) instrument. The structure morphologies of the catalyst particles were determined *via* the TEM images using an EM10C instrument with an accelerating voltage of 100 kV. TGA-DTA analysis was performed with a heating rate of 30 °C min^−1^ over a temperature range of 25–1200 °C using the PerkinElmer Pyris Diamond apparatus. The BET-BJH analyses were performed by BELSORP MINI II. The ICP measurements were performed using a PerkinElmer 5300 DV ICP/OES. Progress of the reaction progress was monitored by TLC (silica gel SIL G/UV 254 plates), and ultrasonication was performed by ultrasonic device 2200 ETH SONICA.

### 2.3. Synthesis of E

E was synthesized as follows:

#### 2.3.1. Stage one: preparation of TA-Olig (A)

A homogeneous solution of polyethylene glycol (PEG-500, 2 mL) and ammonia (25%, 0.5 mL) in (H_2_O : EtOH 5 : 1) was prepared in 1 hour at room temperature. Then, TA (1 g) was added to the solution. After homogenization (about 30 min), a saturated solution of CH_2_O in water (0.3 mL) was added and stirred for 24 hours. Then, A was autoclaved for 24 at 120 °C, centrifuged, and dried at 80 °C.^3^

#### 2.3.2. Stage two: preparation of Ce–Mn-LDH (B)

Solution number one was prepared by dissolving Ce(NO_3_)_3_·6H_2_O (2.6 g, 7 mmol) and Mn(NO_3_)_2_·4H_2_O (7.02 g, 28 mmol) (molar ratio: 1 : 4) in water (50 mL). Solution number two was prepared by dissolving Na_2_CO_3_ (10.6 g, 0.1 mol) and NaOH (4 g, 0.1 mol) (molar ratio: 1 : 1) in water (50 mL), heated at 60 °C for 24 h and added dropwise to solution number one until the pH of the new solution reached 10. Then, the prepared dark brown solid (B) was separated, washed several times with water, and oven-dried at 80 °C.

#### 2.3.3. Stage three: preparation of Ce–Mn-LDH@CPTMS (C)

B (1.0 g) was dispersed in toluene (50 mL) with ultrasonication for 15 min and (3-chloro-propyl)trimethoxysilane (CPTMS) (11 mmol, 2.0 mL) was added dropwise and the mixture refluxed at 110 °C for 12 h. C was collected and washed with toluene and ethanol several times and dried at 80 °C.

#### 2.3.4. Stage four: preparation of Ce–Mn-LDH@CPTMS@TA-Olig (D)

C (1 g) was dispersed in the H_2_O : EtOH (5 : 1) mixture by ultrasonication for about 10 min. A (0.5 g) was also dispersed in H_2_O : EtOH (ratio 5 : 1) by ultrasonication for about 10 min, and was then added dropwise to C, refluxed at 110 °C for 48 h, and D separated by centrifugation and dried at 80 °C.

#### 2.3.5. Stage five: preparation of Ce–Mn-LDH@CPTMS@TA-Olig@Co (E)

D (1 g) was dispersed in H_2_O : EtOH (5 : 1) by ultrasonication for 10 min and Co(NO_3_)_2_·6H_2_O (0.29 g, 5 mmol) was added. The mixture refluxed at 110 °C for 24 h, autoclaved at 120 °C for 24 h, and E was collected and washed with H_2_O/EtOH several times and dried at 80 °C.

### 2.4. General procedure for the synthesis of a(1–12)

A mixture of 5-amino-tetrazole (85 mg, 1 mmol), aldehyde (1 mmol), 1,3-diketone (280 mg, 1 mmol), and E (10 mg) was stirred in solvent-free condition at 70 °C for the specified time. After completion of the reaction (TLC, *n*-hexane/acetone 5 : 3), the mixture was cooled to room temperature, and hot ethanol (5 mL) was added to dissolve products. E was easily separated from the ethanolic solution by centrifugation, the product was washed with ethanol (3 × 20 mL), dried under reduced pressure, and characterized by comparing their IR, NMR spectra, and melting points with authentic samples.^26^

## 3. Results and discussion

### 3.1. Characterization of the catalyst

E was characterized by Fourier-Transform Infrared (FT-IR), X-ray Diffraction (XRD), Energy Dispersive X-ray (EDX), EDX-Mapping, Scanning Electron Microscopy (SEM), Transmission Electron Microscopy (TEM), Thermo-Gravimetric-Differential Thermal Analysis (TGA-DTA), Brunauer–Emmett–Teller (BET)–Barrett–Joyner–Halenda (BJH) and Inductively Coupled Plasma-Optical Emission Spectroscopy (ICP-OES) techniques.

#### 3.1.1. Characterization by FT-IR

**Fig. 1:**
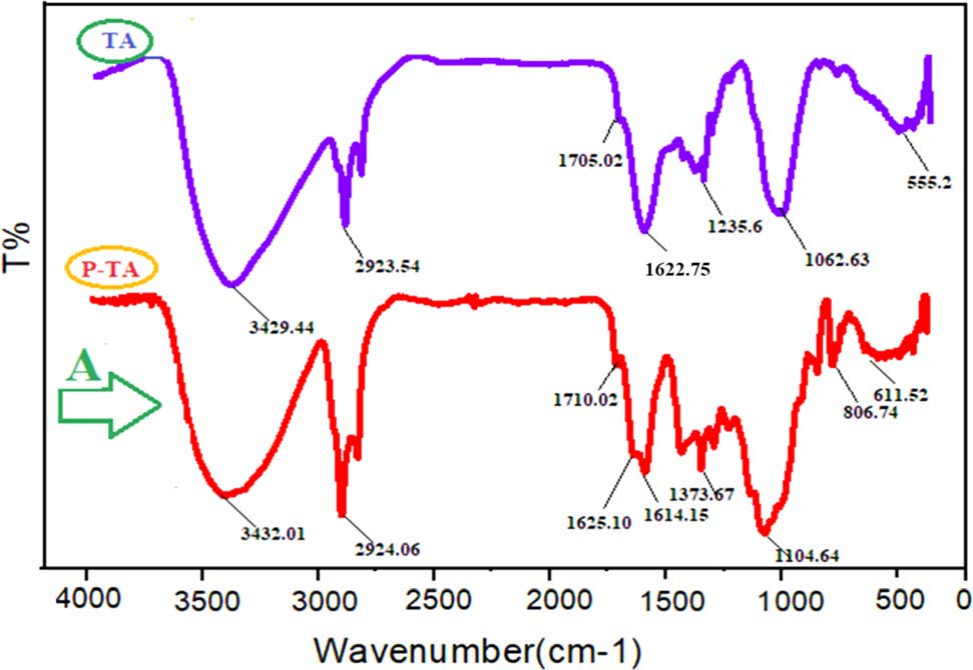
Comparison of the FT-IR spectra of TA, and A.

Fig. 1 shows the FT-IR spectra of TA and TA-Olig. In curve A, new peaks at 806, 611, 1104, and 1614 cm^−1^ confirm the synthesis of TA-Olig.

**Fig. 2:**
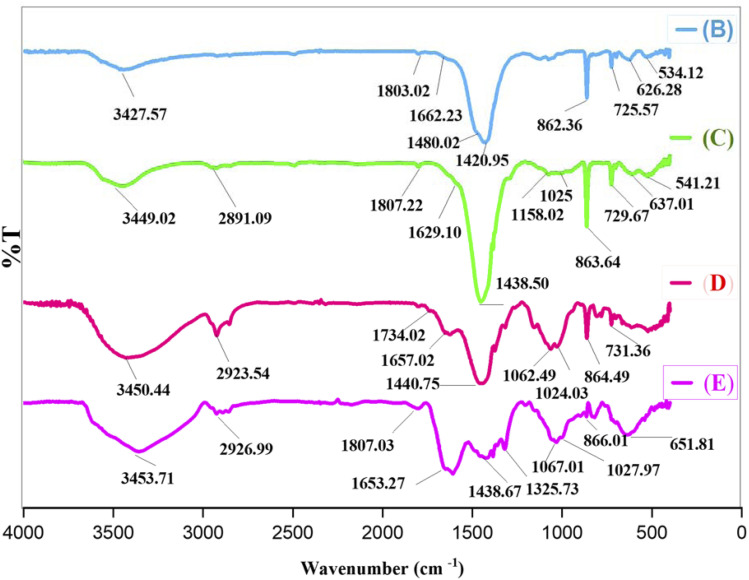
Comparison of the FT-IR spectra of B, C, D, and E.

Fig. 2 represents the FT-IR spectra of B, C, D, and E.

Curve B exhibits three peaks at 626, 534, and 862 cm^−1^, corresponding to Ce–O, Mn–O, and Ce–O–Mn bonds, respectively. The broad peak at about 3427 cm^−1^ is attributed to the OH stretching vibrations, and the peak at 1662 cm^−1^ is associated with the bending vibrations of interlayer water. Additionally, the peaks at 1803 and 1480 cm^−1^ are linked to stretching and asymmetric vibrations of C–O and C=O bonds, respectively.

Curve C shows peaks at 1025 and 1158 cm^−1^, indicative of Si–O–M and Si–O–Si bonds.

In curve D, the shift of peaks to 1657 and 1734 cm^−1^, as well as the broadening of the peak at 3449 cm^−1^, indicates the functionalization of C with A.

In curve E, the presence of a Co–O peak at about 651 cm^−1^, confirms the successful synthesis of E.

#### 3.1.2. Characterization by XRD

**Fig. 3:**
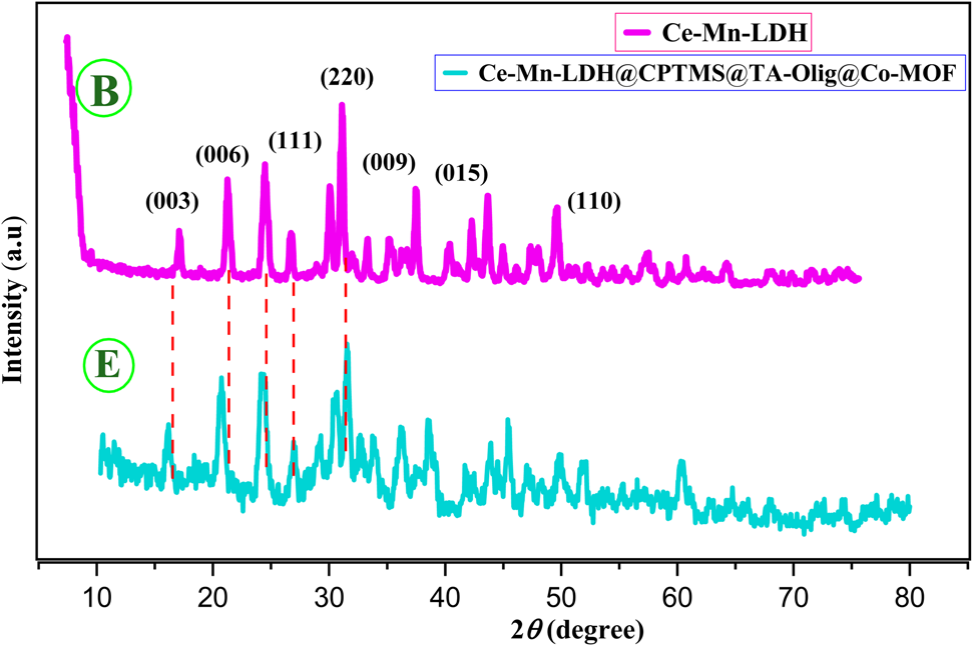
The XRD patterns of B and E.

The XRD structural information of B and E is depicted in Fig. 3. The sharp and symmetrical peaks indicate the structural specifications of hydrotalcite. There are diffraction peaks at 2*θ* = 15.85°, 24.1°, 26.45°, 31.3°, 44.95°, 51.55°, and 52°, corresponding to the (003), (006), (111), (220), (009), (015), and (110) crystal planes of B. The (003) peak shows the degree of crystallinity of structure and the success of B.^32–35^

In graph E, the peak intensities are diminished, and the emergence of peaks at around 41.5°, 44.2°, and 47.3° shows the formation of Co-MOF (Co-PTA). The XRD pattern of the E composite indicates the simultaneous presence of both B and E in the composite structure and the growth of Co-MOF (Co-PTA) crystals on the Ce–Mn-LDH sheets.^36^

#### 3.1.3. Characterization by FESEM-EDX-mapping

**Fig. 4:**
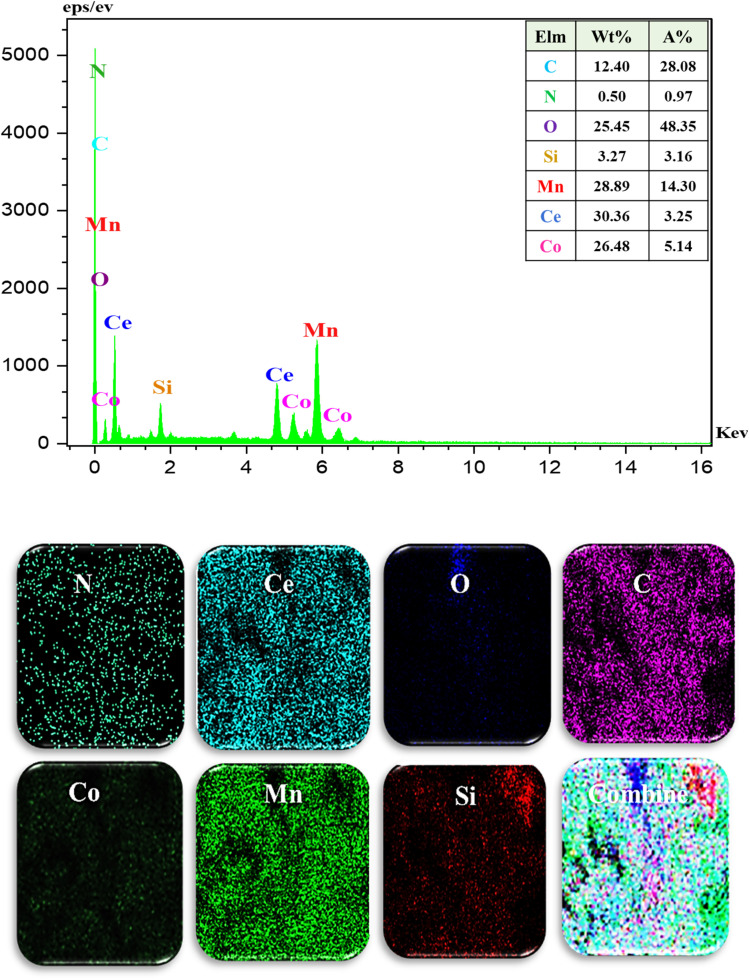
The FESEM-EDX-mapping images of E.

The SEM-EDX mapping of E and the corresponding structural elements are shown in Fig. 4. EDX content analysis confirms the presence of Ce, Mn, O, N, Co, Si, and C elements and FESEM-mapping shows that the LDH/MOF has a uniform surface structure with a homogeneous distribution of these elements. It means that the Co-MOF (Co-PTA) nanocrystals are uniformly distributed on the LDH ultrathin sheets.

#### 3.1.4. Characterization by the SEM images

**Fig. 5:**
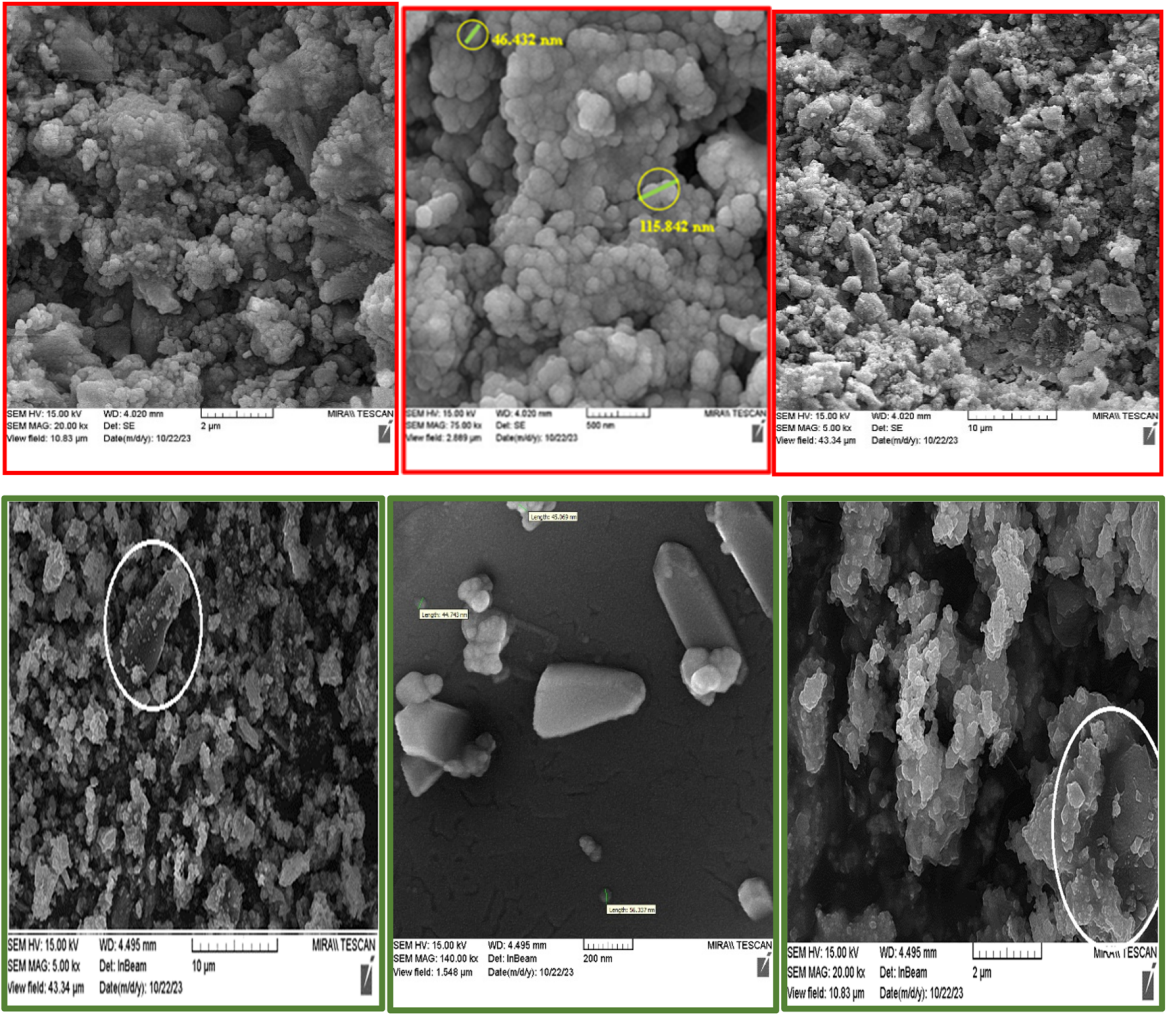
The SEM images of A and E.

The morphology of A and E was confirmed by the SEM images as shown in Fig. 5 (first row). The spherical morphology suggests that the polymerization process was controlled and consistent, and the spherical shape and the average particle size are estimated to be in the nanometer range (86–115 nm), which is a confirmation of the polymerization of TA. Nano-particles can sometimes aggregate, forming larger clusters or blocks, which can occur during sample preparation or due to interactions between the nanoparticles and the LDH sheets.

The FESEM images (Fig. 5, second row) clearly show the presence and successful growth of Co-MOF (Co-PTA) nanoparticles on surface of the Ce–Mn-LDH ultrathin nanosheets.

#### 3.1.5. Characterization by the TEM images

**Fig. 6:**
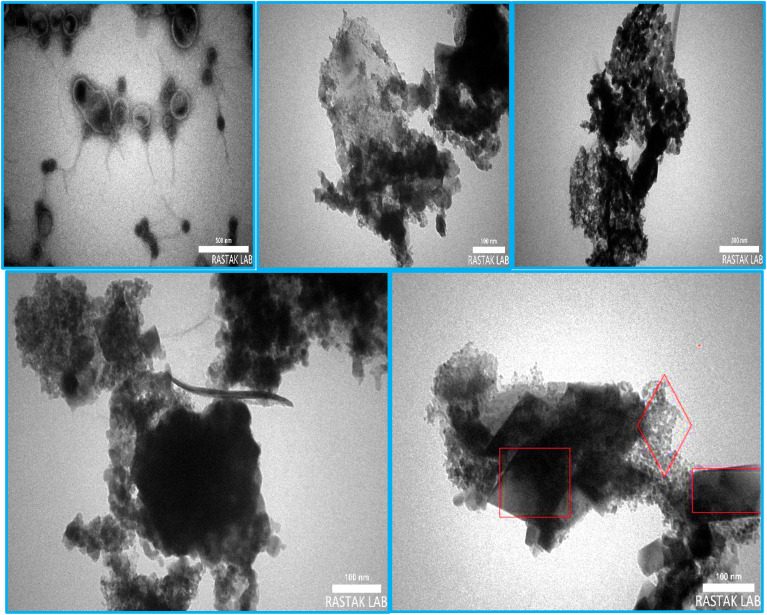
The TEM images of E.

The crystalline structure and morphology of Ce–Mn-LDH@CPTMS@TA-Olig@Co-MOF were studied by the TEM images (Fig. 6). Accordingly, the core–shell structure and the arrangement of metal spheres Co-MOF (Co-PTA) on the Ce–Mn-LDH sheets are visible.

#### 3.1.6. Characterization by TGA-DTA

**Fig. 7:**
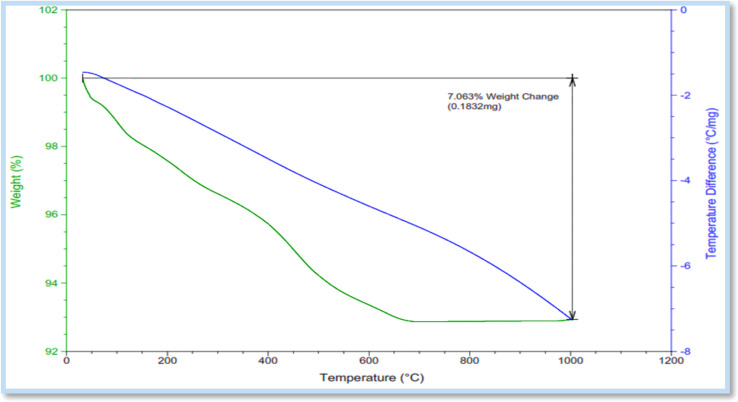
The TGA-DTA curve of E.

The thermal behavior of E was determined by the TGA-DTA curves in the temperature range of 25 to 1000 °C. The gradual weight loss of the catalyst starts at about 75 °C and continues until about 700 °C. This gradual decrease in catalyst weight at around 75 °C can be attributed to the removal of molecules that do not have a strong bond with the main skeleton of the catalyst, or possibly the start of removal of water molecules. The weight loss at about 266 to 400 °C is related to the decomposition of the organic part of the composite (probably water molecules and organic solvents trapped in the pores and cavities of the catalyst). From 400 to 700 °C, with continuous loss of weight (7% weight), the thermal decomposition of different parts of E occurs (Fig. 7).

#### 3.1.7. Characterization by BET/BJH

**Fig. 8:**
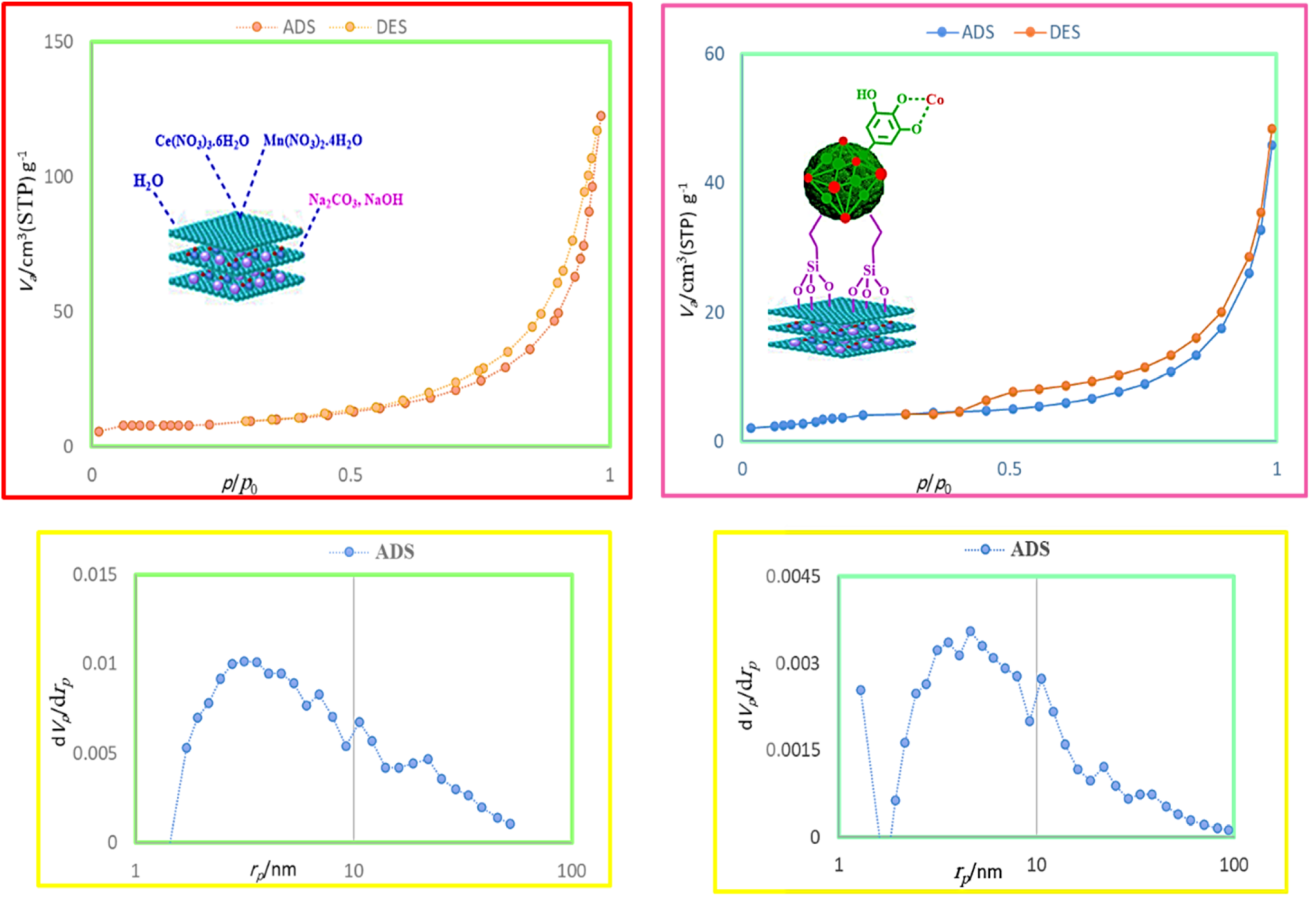
The BET and BJH analyses of B and E.

The textural properties of B and E were evaluated through the nitrogen absorption and desorption analyzes (surface area, pore volume, and pore diameter). As shown in Table 1 and Fig. 8, the surface area decreases from B (27.833 m^2^ g^−1^) to E (20.878 m^2^ g^−1^), indicating that the growth of Co-MOF (Co-PTA) crystals on the surface of B has been carried out, leading to a decrease in the available surface in E.

**Table 1:** The N_2_ absorption–desorption parameters for B and E

	*S*_BET_ (m^2^ g^−1^)	*V*_t_ (cm^3^ g^−1^)	*D*_BJH_ (nm)
B	27.833	0.1899	27.296
E	20.878	0.0701	13.445

The significant decrease in pore volume from B (0.1899 cm^3^ g^−1^) to E (0.0701 cm^3^ g^−1^), indicates that the pores in B are being filled or blocked by the MOF crystals, which is consistent with the successful synthesis of E.

The decrease in pore diameter in E (13.445 nm) compared to B indicates the formation of Co-MOF (Co-PTA) crystals in the pores of B (27.296 nm), leading to smaller pore sizes.

The E isotherm shows some mesoporosity (pores with diameters between 2 and 50 nm), which is typical for materials with MOF structures. This porosity is probably due to the presence of MOF crystals, which create smaller and more uniform pores.

The B isotherm shows the absence of mesoporosity in B, the pores being larger and more irregular, which is consistent with the observed larger pore diameter.

#### 3.1.8. Characterization by ICP-OES

The ICP analysis was used for measuring the amount of Co in E which was about 30.54 mmol g^−1^.

### 3.2. Optimization

To optimize the synthesis of 10a, the one-pot condensation reaction of 5-amino-tetrazole, 4-chlorobenzaldehyde, and dimedone was carried out in various temperatures, catalyst amounts, and solvents in the presence of E. The best optimal conditions were about 1 : 1 : 1 mole of 5-aminotetrazole, 4-chlorobenzaldehyde, and dimedone with 10 mg of E at 70 °C under solvent-free conditions (Table 2).

**Table 2:** Optimization of the reaction conditions for preparation of 10a by E^a^

Entry	Amount of catalyst	Solvent	Temp (°C)	Time (min)	Yield^b^ (%)
1	10 mg	EtOH	r.t.	45	70
2	10 mg	CHCl_3_	r.t.	60	40
3	10 mg	CH_2_Cl_2_	r.t.	80	35
4	10 mg	CH_3_CN	r.t.	70	54
5	10 mg	Solvent-free	r.t.	30	50
6	10 mg	EtOH	Reflux	20	80
7	10 mg	CHCl_3_	Reflux	60	68
8	10 mg	CH_2_Cl_2_	Reflux	70	58
9	10 mg	CH_3_CN	Reflux	25	80
10	10 mg	Solvent-free	60	20	85
**11**	**10 mg**	**Solvent-free**	**70**	**5**	**90**
12	10 mg	Solvent-free	80	10	88
13	10 mg	Solvent-free	90	10	88
14	20 mg	Solvent-free	70	30	71
15	30 mg	Solvent-free	70	25	77
16	No catalyst	Solvent-free	70	120	65

a Conditions: 5-aminotetrazol (1 mmol), 4-chlorobenzaldehyde (1 mmol), dimedone (1 mmol), solvent-free.

b Isolated pure yield.

### 3.3. Synthesis of a(1–12)

Based on the obtained results from the model reaction (1 : 1 : 1 molar ratio of 5-amino-tetrazole, 4-chlorobenzaldehyde, and dimedone, in ethanol with 10 mg of E at 70 °C), all synthesized compounds were characterized and approved with comparison of their spectroscopic data and melting points with authentic samples (Table 3).

**Table 3:** Synthesis of a(1–12) by E^a^

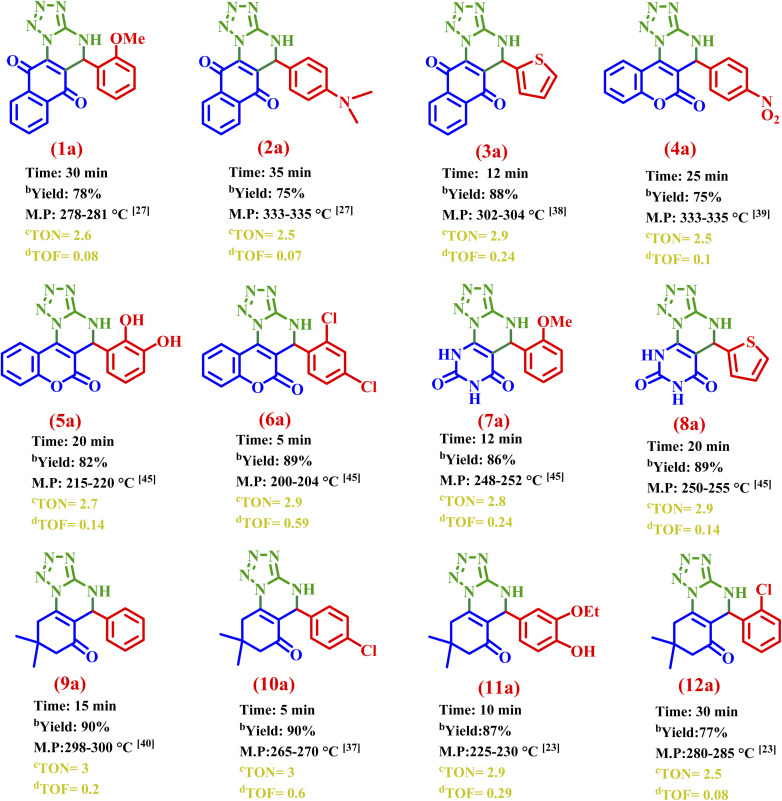

a Reaction conditions: 5-amino tetrazole (1 mmol), 4-chloroaldehyde (1 mmol), dimedone (1 mmol), E (10 mg) in solvent-free condition, at 70 °C.

b Isolated yield.

c TON (turnover number): based on the ICP measurements at a ratio of 1 : 4, the content of Co in the catalyst is about 30.54, so the mmole of Co in 10 mg of the catalyst is 0.3 mmole g^−1^. For entry 1, since the yield is about 78%, the effective mmole the TON is 0.78 ÷ 0.3 = 2.6.

d TOF (turnover frequency): TOF = TON ÷ time. For entry 1, since the time is about 30 min, the TOF is 2.6 ÷ 30 = 0.08.

### 3.4. Spectral data of a(1–12)

#### 3.4.1. 5-(2-Methoxyphenyl)-4,5-dihydrobenzo[*g*]tetrazolo[1,5-*a*]quinazoline-6,11-dione (1a)^27^

Yield: 78%, m.p. 278–281 °C; IR: *ν* = 3233.50, 3086, 2928, 1665.66, 1643.82, 1618.56, 1586.63, 1525.10, 1344.61, 1303.11, 1199.80, 724.82 cm^−1^. ^1^H NMR (300 MHz, DMSO-*d*_6_) *δ*_H_ = 12.04 (s, 1 NH), 8.08–7.58 (s, ArH), 7.55–7.08 (d, *J* = 7.6 Hz, 2 ArH), 6.94 (d, *J* = 8.0 Hz, 2 ArH), 3.62 (s, 1H, MeOH) ppm. ^13^C NMR (75 MHz, DMSO-*d*_6_) *δ*_C_ = 149.70, 139.71, 135.57, 134.38, 131.99, 130.89, 126.66, 126.38, 120.84, 115.10, 112.83, 112.37, 56.13, 40.97, 40.63, 40.30, 39.97, 39.63, 39.30, 38.96 ppm.

#### 3.4.2. 5-(4-(Dimethylamino)phenyl)-4,5-dihydrobenzo[*g*]tetrazolo[1,5-*a*]quinazoline-6,11-dione (2a)^27^

Yield: 88%, m.p. 333–335 °C; IR: *ν* = 3424.50, 292.81, 1682.09, 1580.12, 1574.06, 130.76, 1259.89, 1022.70, 754 cm^−1^. ^1^H NMR (300 MHz, DMSO-*d*_6_) *δ*_H_ = 12.05 (s, 1 NH), 8.45–7.56 (d, *J* = 8.8 Hz, 2 ArH), 7.28–6.94 (d, *J* = 7.6 Hz, 2 ArH), 5.18 (s, Hz, ArH), 3.62 and 3.52 (s, 2 CH_3_) ppm. ^13^C NMR (75 MHz, DMSO-*d*_6_) *δ*_C_ = 180.45, 179.01, 157.63, 135.56, 135.33, 134.13, 132.54, 131.95, 131.40, 130.82, 126.93, 126.67, 126.15, 125.44, 120.85, 120.69, 112.52, 112.36, 56.10, 55.22, 40.94, 40.61, 40.28, 39.94, 39.61, 39.28, 38.95 ppm.

#### 3.4.3. 5-(Thiophen-2-yl)-4,5-dihydrobenzo[*g*]tetrazolo[1,5-*a*]quinazoline-6,11-dione (3a)^37^

Yield: 80%, m.p. 302–304 °C; IR: *ν* = 338.95, 2923.78, 1682.25, 1597.91, 1573.82, 1375, 1061.7, 474 cm^−1^. ^1^H NMR (300 MHz, DMSO-*d*_6_) *δ*_H_ = 14.47 (s, 1 NH), 7.91–7.06 (d, *J* = 8.4 Hz, 2 ArH), 6.30 (d, *J* = 6.8 Hz, 2 ArH), 5.54 (s, ArH) ppm. ^13^C NMR (75 MHz, DMSO-*d*_6_) *δ*_C_ = 178.61, 172.55, 151.46, 149.99, 144.49, 139.63, 137.03, 134.87, 133.19, 128.64, 126.08, 124.56, 122.80, 121.49, 120.74, 41.30, 21.18 ppm.

#### 3.4.4. 5-(4-Nitrophenyl)-4,5-dihydro-6*H*-chromeno[3,4-*e*]tetrazolo[1,5-*a*]pyrimidin-6-one (4a)^38^

Yield: 90%, m.p. 265–270 °C; IR: *ν* = 3419.5, 3255.67, 3074.36, 2923, 2727, 1666.23, 1614.88, 1563.77, 1523.48, 1491.60, 1453.91, 1313.33, 1266.27, 1215.92, 856.15, 785.94, 682.58 cm^−1^. ^1^H NMR (300 MHz, DMSO-*d*_6_) *δ*_H_ = 10.14 (s, 1 NH), 8.37 (s, ArH), 8.05 (d, *J* = 7.6 Hz, 2 ArH), 7.84 (d, *J* = 8.4 Hz, 2 ArH), 7.55 (m, 2 ArH), 7.38–7.30 (d, *J* = 8.8 Hz, 2 ArH), 6.38 (s, ArH) ppm. ^13^C NMR (75 MHz, DMSO-*d*_6_) *δ*_C_ = 192.74, 167.15, 164.87, 152.88, 132.11, 124.51, 123.87, 123.62, 116.26, 103.64, 40.55, 40.24, 39.90, 39.57, 39.23 ppm.

#### 3.4.5. 5-(2,3-Dihydroxyphenyl)-4,5-dihydro-6*H*-chromeno[3,4-*e*]tetrazolo[1,5-*a*]pyrimidin-6-one (5a)^39^

Yield: 82%, m.p. 215–220 °C; IR: *ν* = 3429.7, 3213.65, 3087.89, 2850.86, 1755.73, 1703.58, 1574.94, 1443.59, 1413.96, 1289.40, 1202.39, 1093.16, 1019.75, 989.51, 838.75, 717.50 cm^−1^. ^1^H NMR (300 MHz, DMSO-*d*_6_) *δ*_H_ = 12.15 (s, 1 OH), 9.84 (s, 1 NH), 8.31–8.01 (s, ArH), 7.65–7.43 (d, *J* = 8.2 Hz, 2 ArH), 6.92–5.69 (d, *J* = 8.4 Hz, 2 ArH), 3.46 (s, 1 OH) ppm. ^13^C NMR (75 MHz, DMSO-*d*_6_) *δ*_C_ = 183.80, 159.64, 149.64, 143.01, 129.08, 128.03, 127.26, 124.59, 119.59, 118.12, 116.13, 114.29, 40.89, 40.56, 40.23, 39.89, 39.56, 39.22, 38.89 ppm.

#### 3.4.6. 5-(2,4-Dichlorophenyl)-4,5-dihydro-6*H*-chromeno[3,4-*e*]tetrazolo[1,5-*a*]pyrimidin-6-one (6a)^39^

Yield: 89%, m.p. 200–204 °C; IR: *ν* = 3417.89, 3332.30, 3216.45, 1674.38, 1651.74, 1589.22, 1454.06, 1374.95, 1265.15, 1143.66, 1049.65, 1000.03, 736.16, 679.84 cm^−1^. ^1^H NMR (300 MHz, DMSO-*d*_6_) *δ*_H_ = 11.02 (s, 1 NH), 7.64 (s, ArH), 7.59–7.34 (d, *J* = 7.4 Hz, 2 ArH), 7.07–7.01 (d, *J* = 7.8 Hz, 2 ArH), 6.94–6.84 (m, 2 ArH) ppm. ^13^C NMR (75 MHz, DMSO-*d*_6_) *δ*_C_ = 184.83, 159.80, 151.16, 138.80, 125.11, 123.19, 118.24, 116.31, 112.64, 40.93, 40.60, 40.27, 39.94, 39.60, 39.27, 38.94 ppm.

#### 3.4.7. 5-(Thiophen-2-yl)-5,9-dihydropyrimido[5,4-*e*]tetrazolo[1,5-*a*]pyrimidine-6,8(4*H*,7*H*)-dione (7a)^39^

Yield: 89%, m.p. 250–255 °C; IR: *ν* = 3424.60, 3202.39, 3086, 2846.70, 1735.27, 1676.74, 1598.43, 1555.99, 1440.71, 1306.36, 1251.44, 1203.68, 1166.33, 1016.03, 795.43, 758.81 cm^−1^. ^1^H NMR (300 MHz, DMSO-*d*_6_) *δ*_H_ = 11.30 (s, 1 NH), 11.26 (s, 1 NH), 8.55–8.14 (s, ArH), 7.33 (s, 1 NH), 3.45, 2.48. ^13^C NMR (75 MHz, DMSO-*d*_6_) *δ*_C_ = 183.78, 159.60, 149.65, 137.70, 132.21, 127.25, 124.56, 119.56, 114.27, 40.93, 40.61, 40.27, 39.94, 39.60, 39.27 ppm.

#### 3.4.8. 5-(2-Methoxyphenyl)-5,9-dihydropyrimido[5,4-*e*]tetrazolo[1,5-*a*]pyrimidine-6,8(4*H*,7*H*)-dione (8a)^39^

Yield: 86%, m.p. 248–252 °C; IR: *ν* = 3427.20, 3203.10, 3152.20, 3054.55, 2853.10, 1751.15, 1696.89, 1654.66, 1549.88, 1395.94, 1233.70, 1062.40, 860.16 cm^−1^. ^1^H NMR (300 MHz, DMSO-*d*_6_) *δ*_H_ = 11.34 (s, 1 NH), 11.15 (s, 1 NH), 8.48–7.49 (s, ArH), 7.46–6.99 (d, *J* = 7.6 Hz, 2 ArH), 6.96 (d, *J* = 7.8 Hz, 2 ArH), 6.93(s, 1 NH), 3.86 (s, 1H, MeOH) ppm. ^13^C NMR (75 MHz, DMSO-*d*_6_) *δ*_C_ = 163.88, 161.91, 159.46, 150.34, 134.57, 132.91, 121.94, 119.92, 111.41, 56.34, 40.58, 40.25, 39.91, 39.58, 39.25, 38.92 ppm.

#### 3.4.9. 8,8-Dimethyl-5-phenyl-5,7,8,9-tetrahydrotetrazolo[1,5-*a*]quinazolin-6(4*H*)-one (9a)^40^

Yield: 90%, m.p. 298–300 °C; IR: *ν* = 3429.7, 3241.3, 3170.88, 3062.29, 2933.67, 1651.97, 1580.85, 1552.45, 1422.70, 1457.27, 1367.34, 1325.52, 1225.25, 986.95, 843.23, 734.72 cm^−1^. ^1^H NMR (300 MHz, DMSO-*d*_6_) *δ*_H_ = 11.59 (s, 1 NH), 7.34–7.28 (m, 5 ArH), 6.70 (s, 9-CH), 2.58 (s, CH_2_), 2.14 (m, CH_2_), 2.08, and 0.98 (s, 2 CH_3_) ppm. ^13^C NMR (75 MHz, DMSO-*d*_6_) *δ*_C_ = 193.46, 150.93, 148.91, 140.90, 137.57, 129.03, 127.60, 106.11, 57.89, 50.25, 40.63, 40.31, 39.97, 39.64, 39.31, 32.74, 28.70, 27.44 ppm.

#### 3.4.10. 5-(4-Chlorophenyl)-8,8-dimethyl-5,7,8,9-tetrahydrotetrazolo[1,5-*a*]quinazolin-6(4*H*)-one (10a)^41^

Yield: 90%, m.p. 265–270 °C; IR: *ν* = 3434.80, 3246.40, 3177.70, 3057.58, 2960.84, 1652.58, 1584.56, 1468.23, 1430.71, 1369.54, 1338.26, 1228.17, 1051.61, 985.62, 847.08, 759.73, 562.77 cm^−1^. ^1^H NMR (300 MHz, DMSO-*d*_6_) *δ*_H_ = 11.67 (s, 1 NH), 7.43 (d, *J* = 7.8 Hz, 2 ArH), 7.41–7.28 (d, *J* = 7. 8 Hz, 2 ArH), 6.89 (s, 9-CH), 2.57–2.06 (m, CH_2_), 1.05, and 1.00 (s, 2 CH_3_) ppm. ^13^C NMR (75 MHz, DMSO-*d*_6_) *δ*_C_ = 193.41, 151.61, 149.01, 137.39, 130.56, 127.90, 105.05, 56.27, 50.24, 40.95, 40.62, 39.61, 39.28, 38.95, 28.70, 27.93 ppm.

#### 3.4.11. 5-(3-Ethoxy-4-hydroxyphenyl)-8,8-dimethyl-5,7,8,9-tetrahydrotetrazolo[1,5-*a*]quin-azolin-6(4*H*)-one (11a)^23^

Yield: 87%, m.p. 225–230 °C; IR: *ν* = 3429.70, 3238.80, 3172.60, 3060.50, 2968.10, 1651.91, 1580.88, 1422.65, 1367.40, 1315.59, 1245.53, 1140.89, 734.64, 702.79 cm^−1^.

#### 3.4.12. 5-(2-Chlorophenyl)-8,8-dimethyl-5,7,8,9-tetrahydrotetrazolo[1,5-*a*]quinazolin-6(4*H*)-one (12a)^23^

Yield: 77%, m.p. 280–285 °C; IR: *ν* = 3456.20, 3209.80, 3154.85, 3064.27, 2959.18, 1634.51, 1562.41, 1491.80, 1417.98, 1304.19, 1235.80, 1139.40, 971.52, 804.29, 697.30 cm^−1^.

### 3.5. Comparison of the catalyst activities

Table 4 shows the comparison of the previous methods (entries 1–10) used for the synthesis of a(1–12) with our proposed method (entry 11). As can be seen, our proposed method has a short time (5 min) with about 90% yield.

**Table 4:** Comparison of E with other catalysts for the synthesis of a(1–12)

Entry	Catalyst	Conditions	Time (min)	Yield (%)	Ref.
1	*p*-TSOH	Solvent-free, 120 °C	2 h	84	26
2	Fe_3_O_4_@C/Ph SO_3_H	H_2_O, 80 °C	1 h	96	27
3	g-C_3_N_4_/NHSO_3_H	i-PrOH, 80 °C	4 h	95	28
4	NaN_3_, Hg(OAc)_2_	HOAc, 100 °C	6 h	67	29
5	AlCl_3_	CH_3_CN, reflux	3 h	92	30
6	I_2_	i-PrOH, reflux	10 min	92	42
7	*p*-TSA	Solvent-free, 70 °C	6 min	88	43
8	MNPs@SiO_2_-Pr ANDSA	EtOH/H_2_O, 100 °C	5 min	94	44
9	PyTFA	μW, 90 °C	25 min	93	45
10	Our catalyst	Solvent-free, 70 °C	5 min	90	This work

### 3.6. Proposed mechanism

**Scheme 3:**
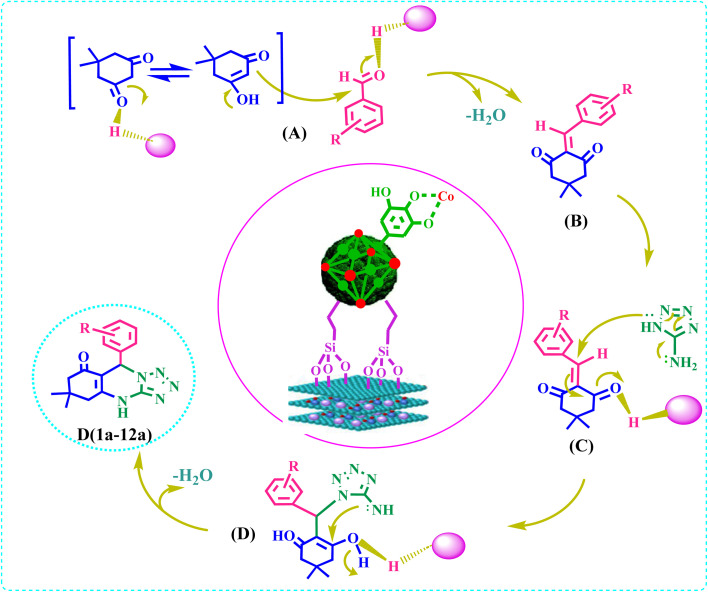
Proposed mechanism for the synthesis of a(1–12).

Scheme 3 shows the possible mechanism for the synthesis of a(1–12) by E.^43,44^

### 3.7. Recyclability of E

**Fig. 9:**
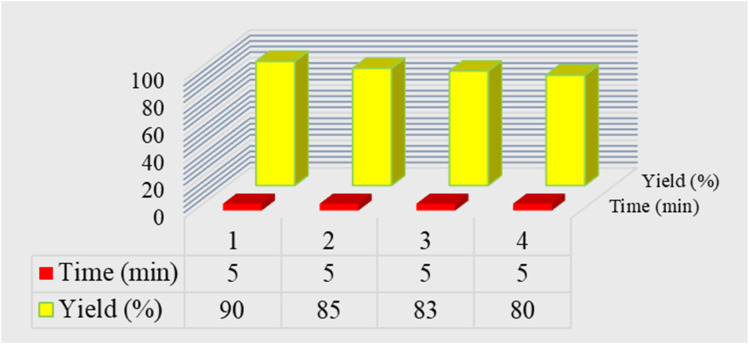
Recyclability test of E.

The recovery ability of the E was investigated in the model reaction after four consecutive runs (Fig. 9). At the end of the reaction, E was washed with water and ethanol, dried at 80 °C, and reused in the next runs. As a result, no significant loss of activity was observed.

**Fig. 10:**
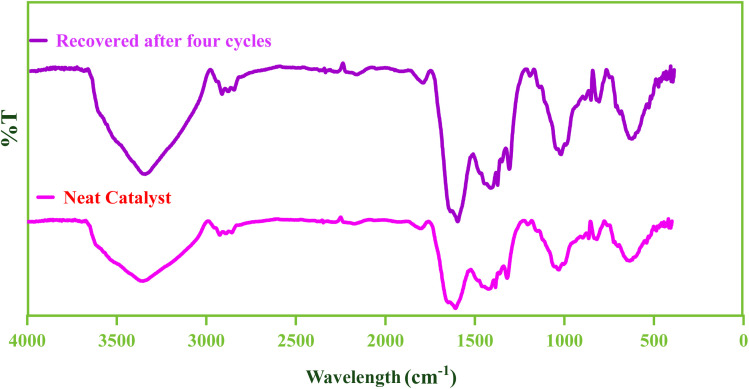
The FT-IR spectra of the fresh and the used E.

In addition, the FT-IR spectrum of E before and after the consecutive runs of recovery showed a very nice similarity (Fig. 10).

## 4. Conclusion

In this study, for the first time, TA oligomers were used as a green ligand for the synthesis of MOFs to modify the surface of the Ce–Mn-LDH. These oligomers have many active sites for chelation with metal by creating metal-phenol-coordinated polymers. Also, FT-IR, ICP, XRD, BET, EDX, SEM, SEM-mapping, TEM, and TGA-DTA techniques showed that the LDH is a suitable surface for the growth of TA oligomers and the formation of metal–organic framework crystals. The catalytic performance of this composite E was evaluated in the synthesis of tetrazolo[1,5-*a*]quinazolines. The high yield of products in a short time and the ability to recycle without side products are the advantages of this composite E.

## Data availability

All data generated or analyzed during this study are included in the ESI.†

## Conflicts of interest

The authors declare that they have no known competing financial interests or personal relationships that could have appeared to influence the work reported in this paper.

## Supplementary Material

NA-007-D4NA00643G-s001
