# Supplementary material for: A new mesoporous Ce–Mn-LDH-based Co-MOF nano-composite for the green synthesis of tetrazoloquinazolines
Source: Nanoscale Adv. 2024 Nov 21;7(4):1077–90. doi: 10.1039/d4na00643g (PMC11668168; doi:10.1039/d4na00643g)
Supplement: NA-007-D4NA00643G-s001 [file NA-007-D4NA00643G-s001.pdf]

Supplementary Information (SI) for Nanoscale Advances.
This journal is © The Royal Society of Chemistry 2024

Supporting Information

(Nanoscale Advances)

A new mesoporous Ce-Mn-LDH-based Co-MOF nano-composite for the green synthesis of tetrazoloquinazolines

Samira Javadi, Davood Habibi*

Department of Organic Chemistry, Faculty of Chemistry and Petroleum Sciences, Bu-Ali Sina University, Hamedan, Iran
*Corresponding author email: davood.habibi@gmail.com (& dhabibi@basu.ac.ir), Tel: 98 81-38380922; Fax: 98 81-31408025

CONTENT

PAGE

IR Spectrum of (**1a**) 2
^{1}HNMR Spectrum of (**1a**) 2
^{13}CNMR Spectrum of (**1a**) 3
IR Spectrum of (**2a**) 3
^{1}HNMR Spectrum of (**2a**) 4
^{13}CNMR Spectrum of (**2a**) 4
IR Spectrum of (**3a**) 5
^{1}HNMR Spectrum of (**3a**) 5
^{13}CNMR Spectrum of (**3a**) 6
IR Spectrum of (**4a**) 6
^{1}HNMR Spectrum of (**4a**) 7
^{13}CNMR Spectrum of (**4a**) 7
IR Spectrum of (**5a**) 8
^{1}HNMR Spectrum of (**5a**) 8
^{13}CNMR Spectrum of (**5a**) 9
IR Spectrum of (**6a**) 9
^{1}HNMR Spectrum of (**6a**) 10
^{13}CNMR Spectrum of (**6a**) 10
IR Spectrum of (**7a**) 11
^{1}HNMR Spectrum of (**7a**) 11
^{13}CNMR Spectrum of (**7a**) 12
IR Spectrum of (**8a**) 12
^{1}HNMR Spectrum of (**8a**) 13
^{13}CNMR Spectrum of (**8a**) 13
IR Spectrum of (**9a**) 14
^{1}HNMR Spectrum of (**9a**) 14
^{13}CNMR Spectrum of (**9a**) 15
IR Spectrum of (**10a**) 15
^{1}HNMR Spectrum of (**10a**) 16
^{13}CNMR Spectrum of (**10a**)
IR Spectrum of (**11a**) 17
IR Spectrum of (**12a**) 17

Spectral data

5-(2-methoxyphenyl)-4,5-dihydrobenzo[*g*]tetrazolo[1,5-*a*] quinazoline-6,11-dione **(1a)**

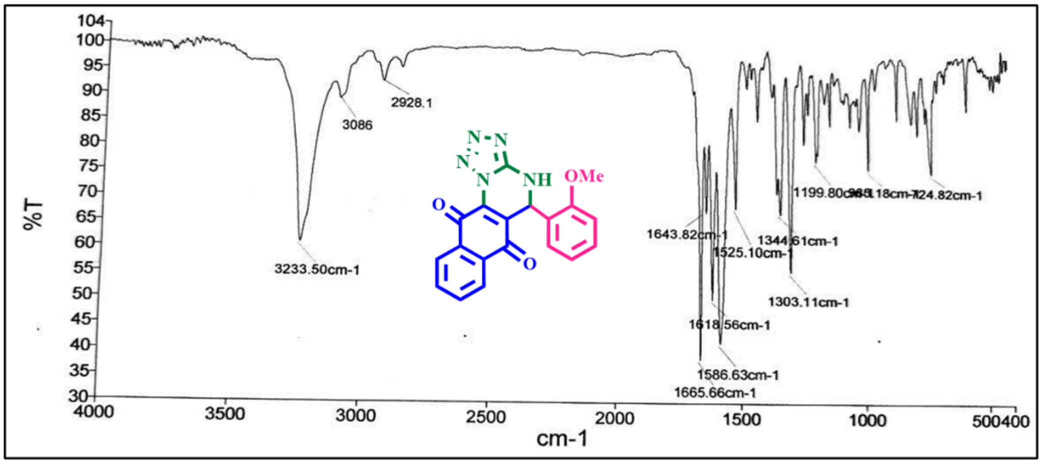

IR Spectrum of 1a

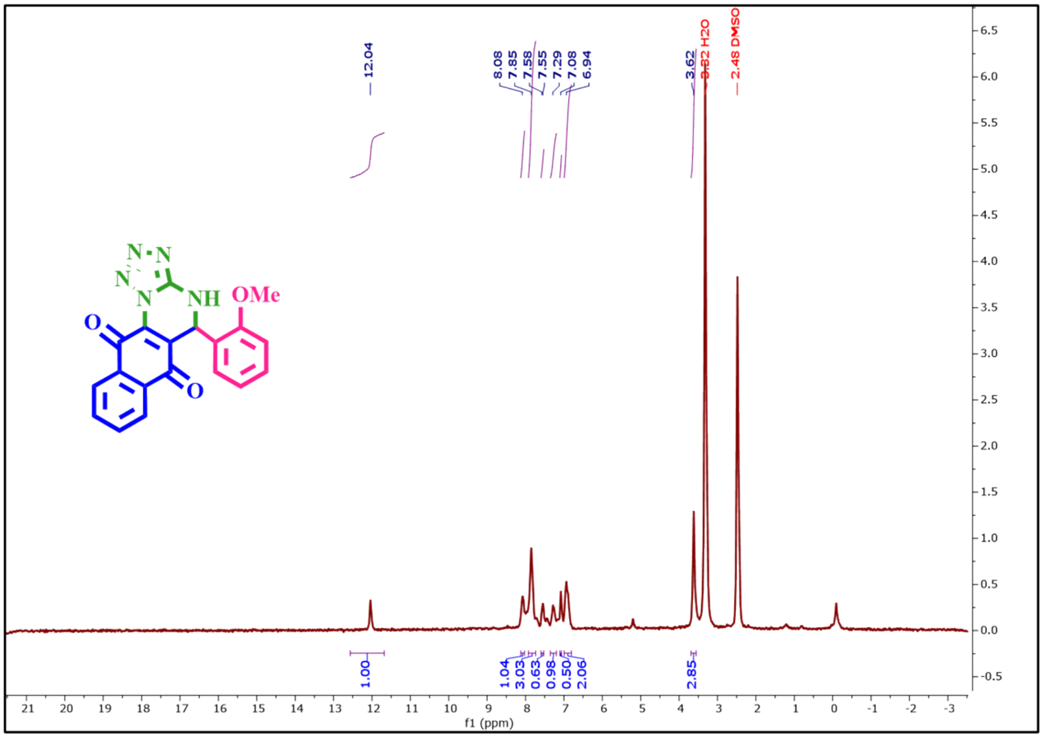

^{1}HNMR Spectrum of 1a

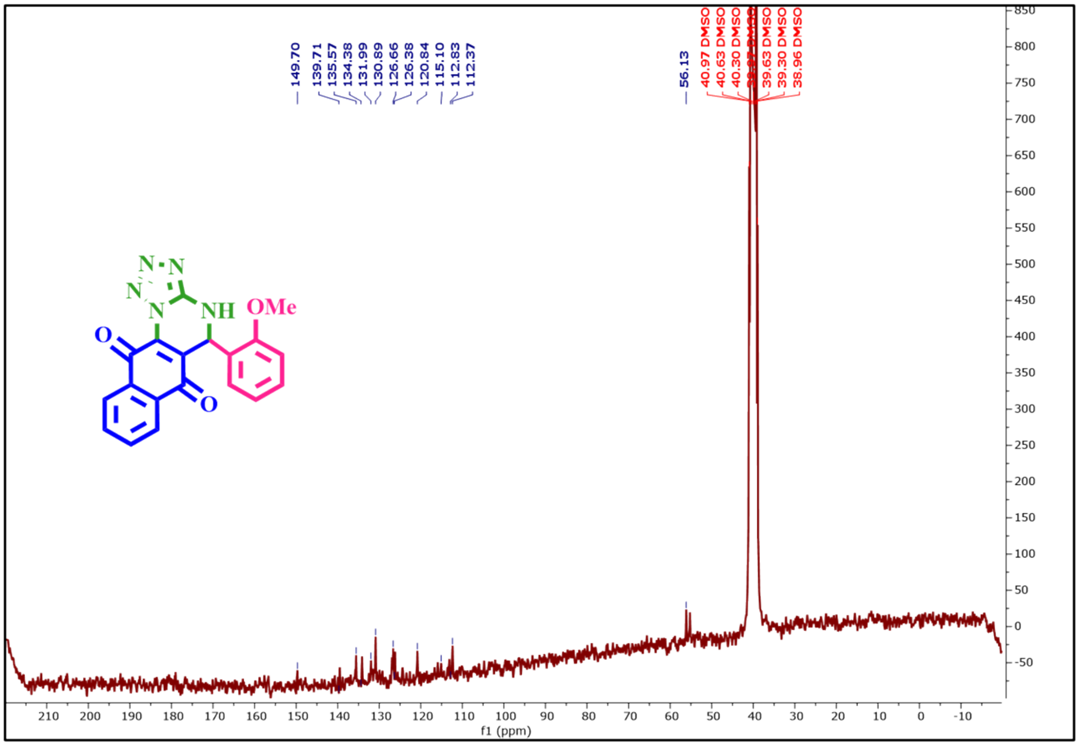

^{13}CNMR Spectrum of 1a

5-(4-(dimethylamino) phenyl)-4,5-dihydrobenzo[*g*]tetrazolo[1,5-*a*] quinazoline-6,11-dione **(2a)**

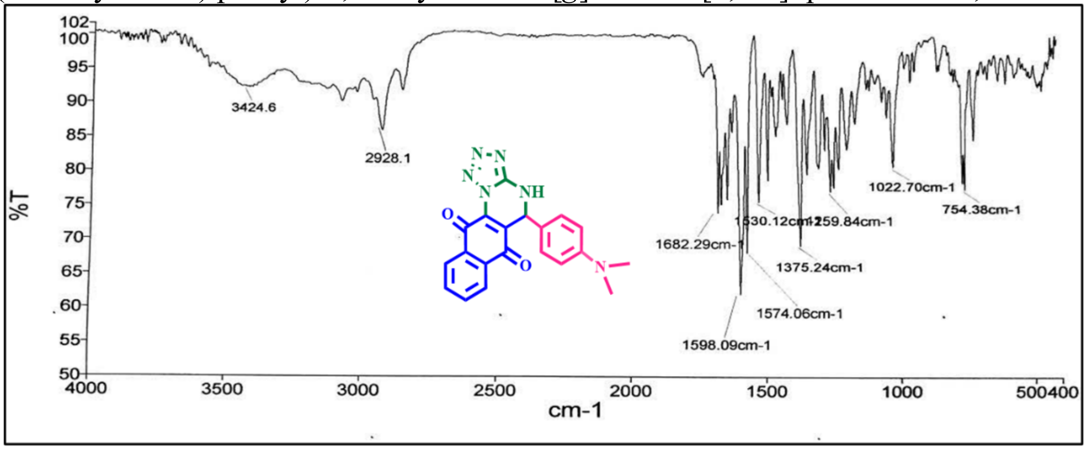

IR Spectrum of 2a

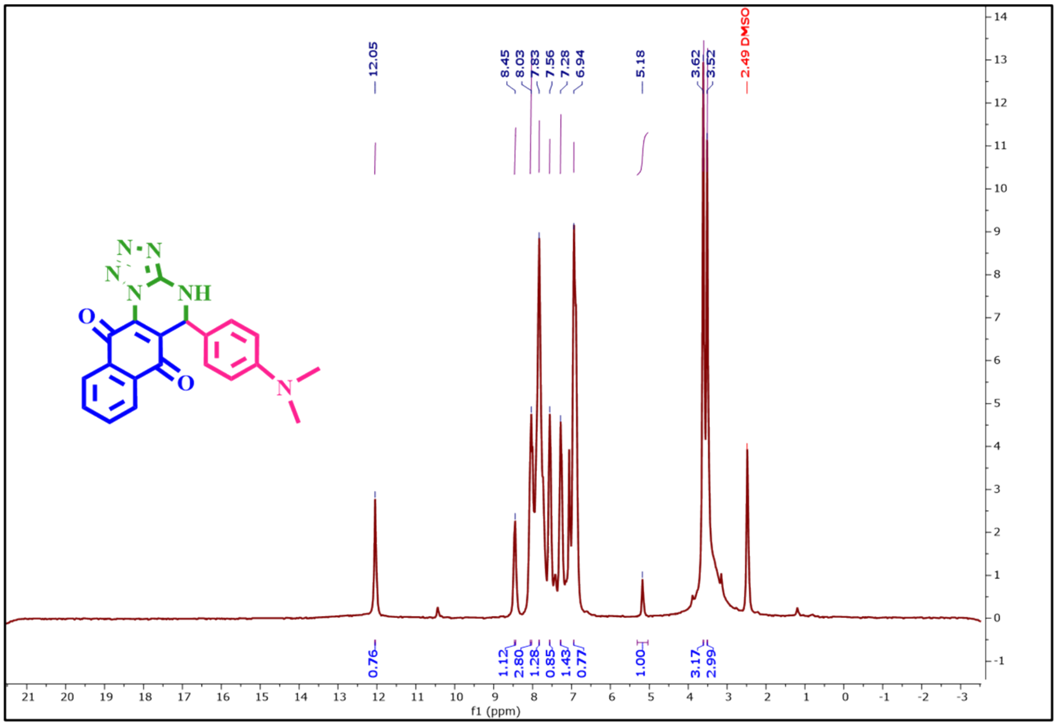

^{1}HNMR Spectrum of 2a

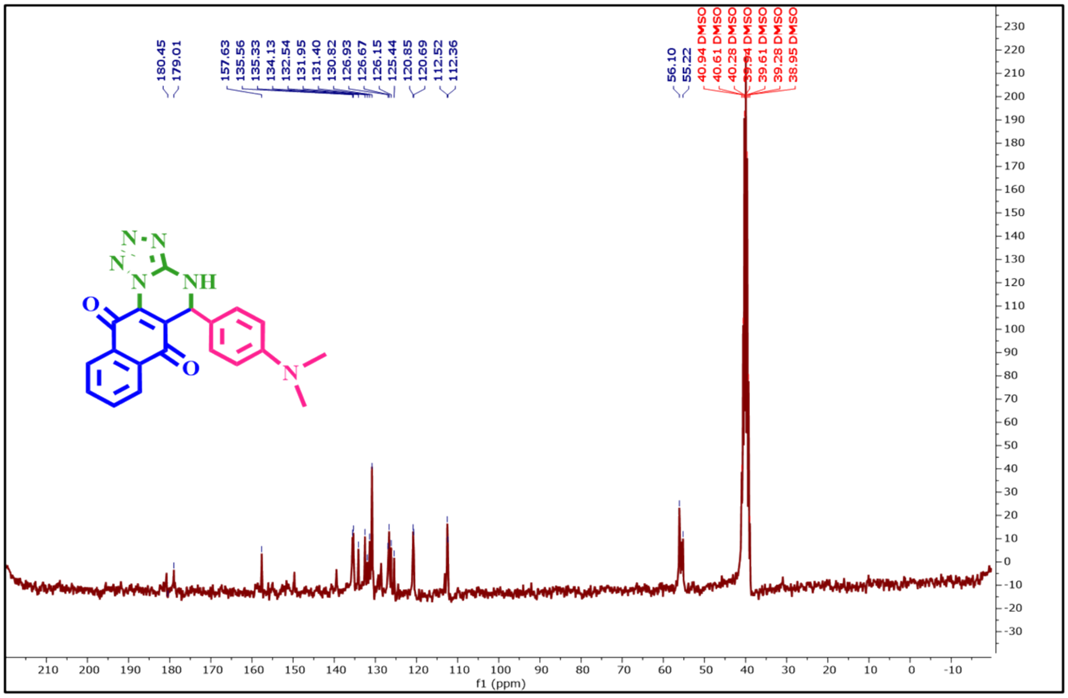

^{13}CNMR Spectrum of 2a

5-(thiophen-2-yl)-4,5-dihydrobenzo[*g*]tetrazolo[1,5-*a*] quinazoline-6,11-dione **(3a)**

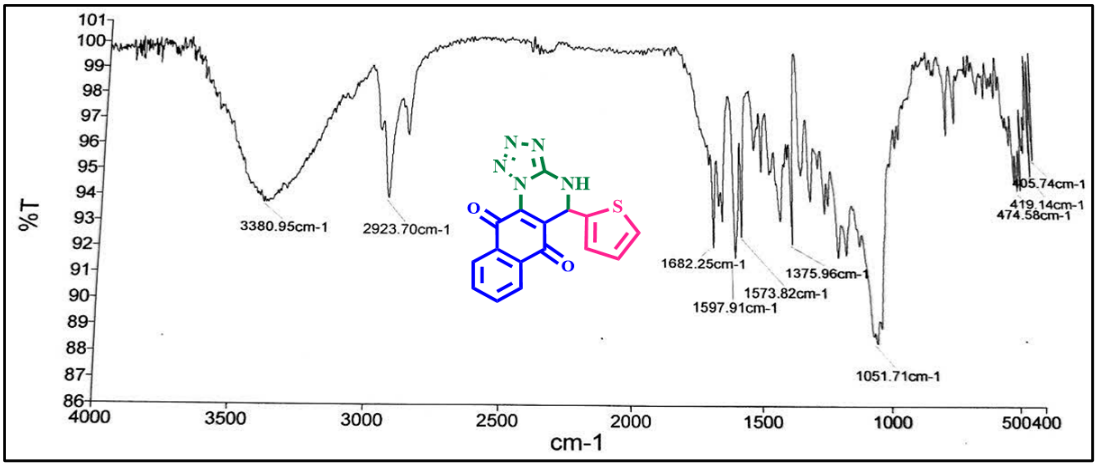

IR Spectrum of 3a

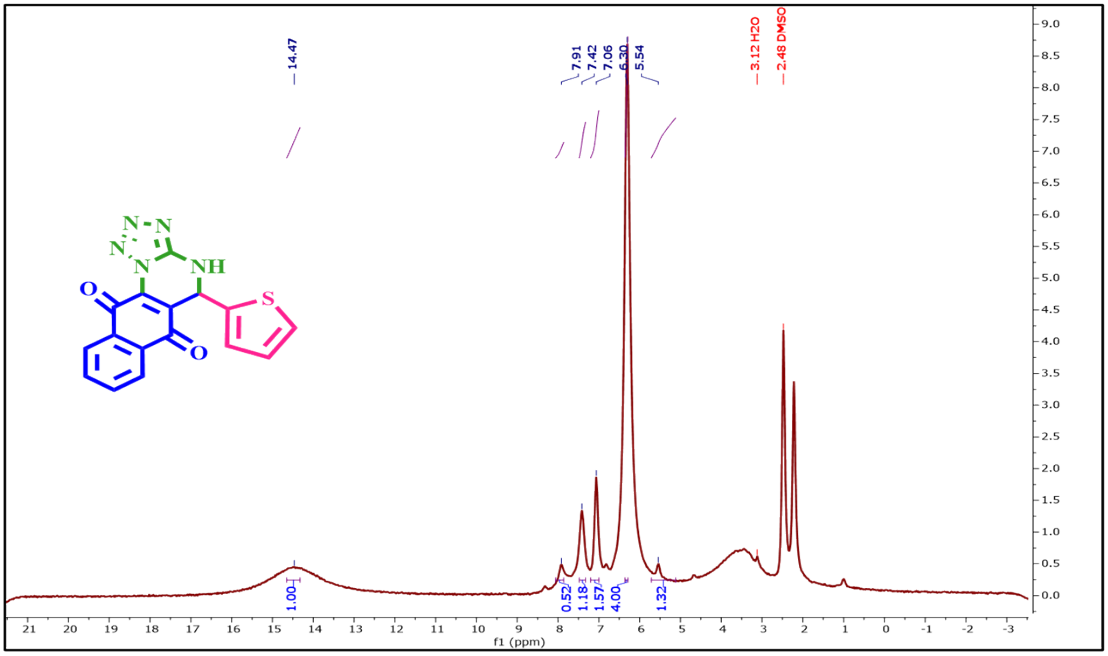

^{1}HNMR Spectrum of 3a

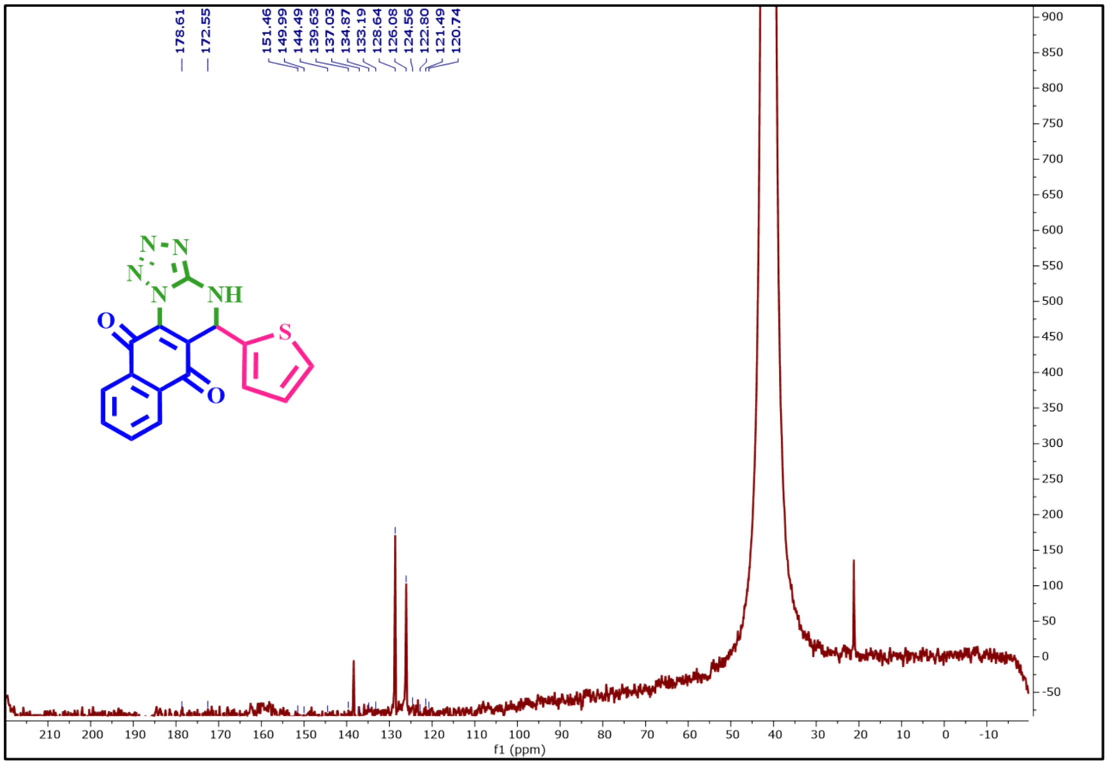

^{13}CNMR Spectrum of 3a

5-(4-nitrophenyl)-4,5-dihydro-6*H*-chromeno[3,4-*e*]tetrazolo[1,5-*a*]pyrimidin-6-one **(4a)**

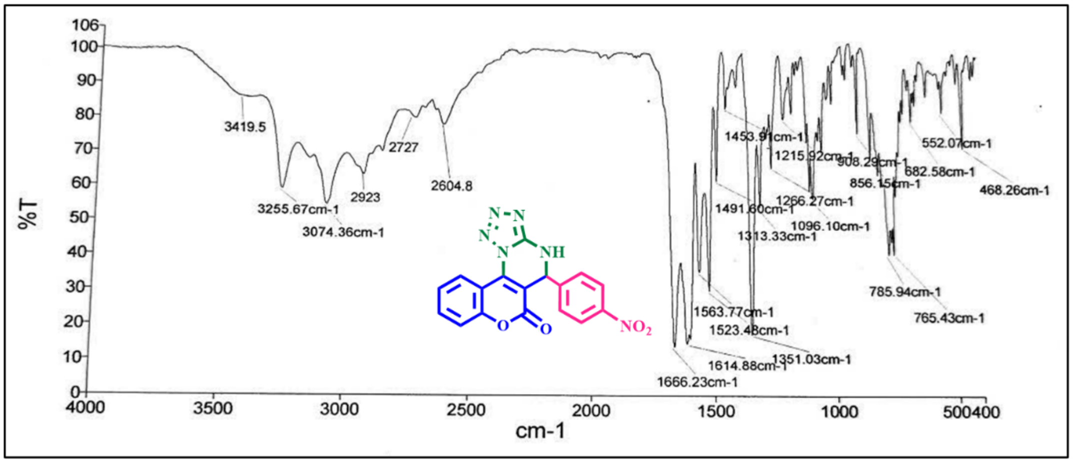

IR Spectrum of 4a

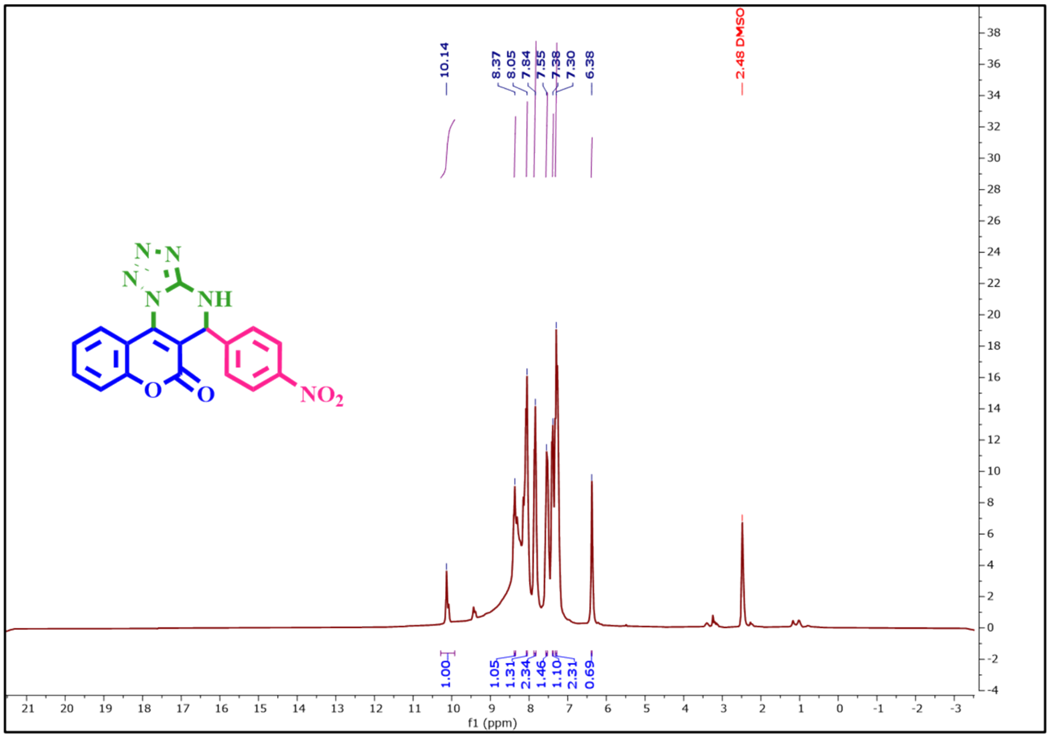

^{1}HNMR Spectrum of 4a

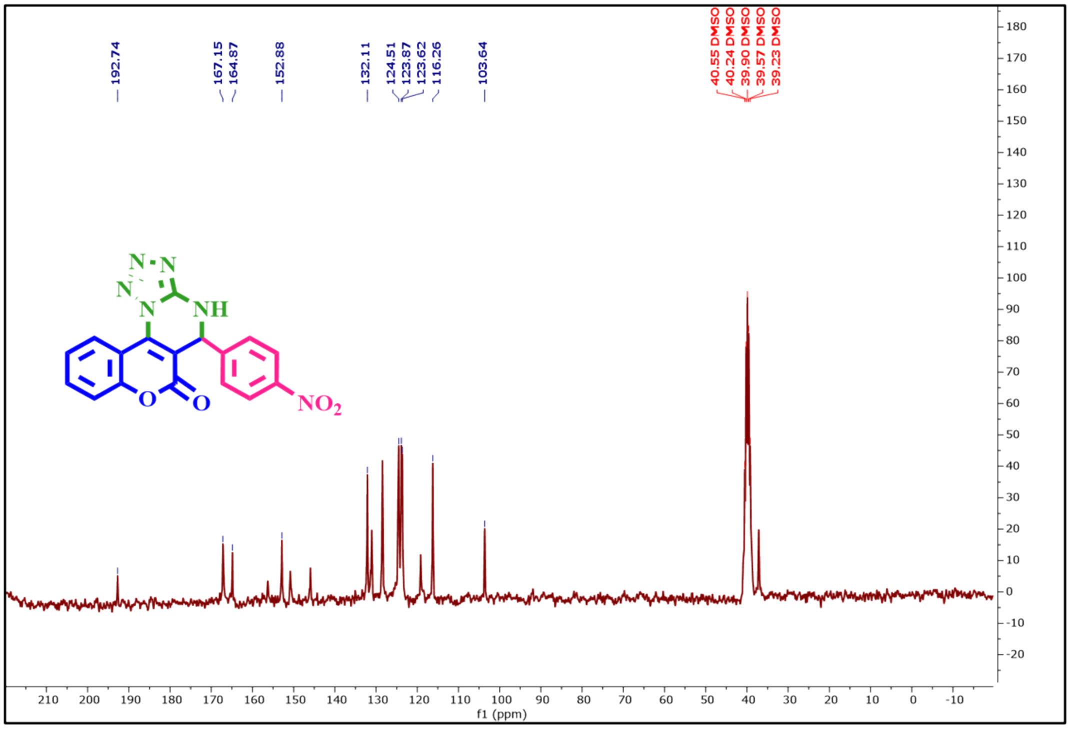

^{13}CNMR Spectrum of 4a

5-(2,3-dihydroxyphenyl)-4,5-dihydro-6*H*-chromeno[3,4-*e*] tetrazolo[1,5-*a*] pyrimidin-6-one **(5a)**

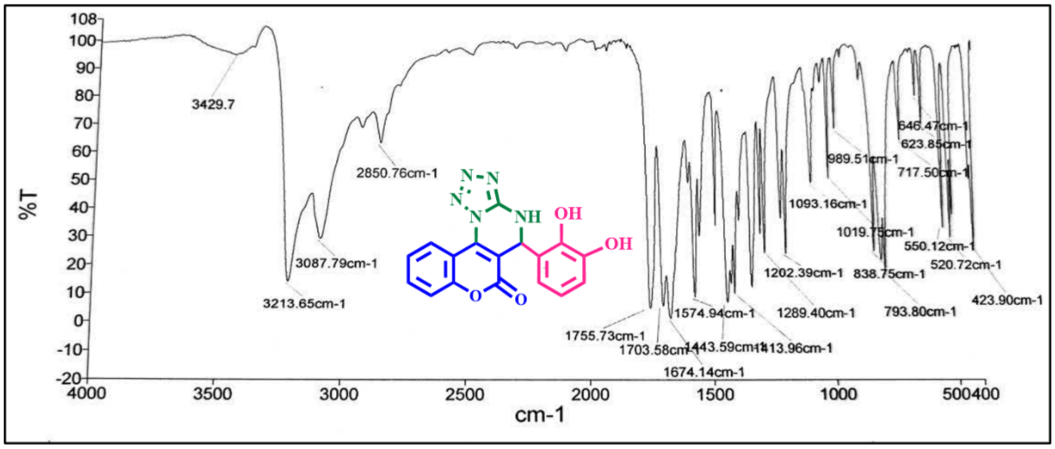

IR Spectrum of 5a

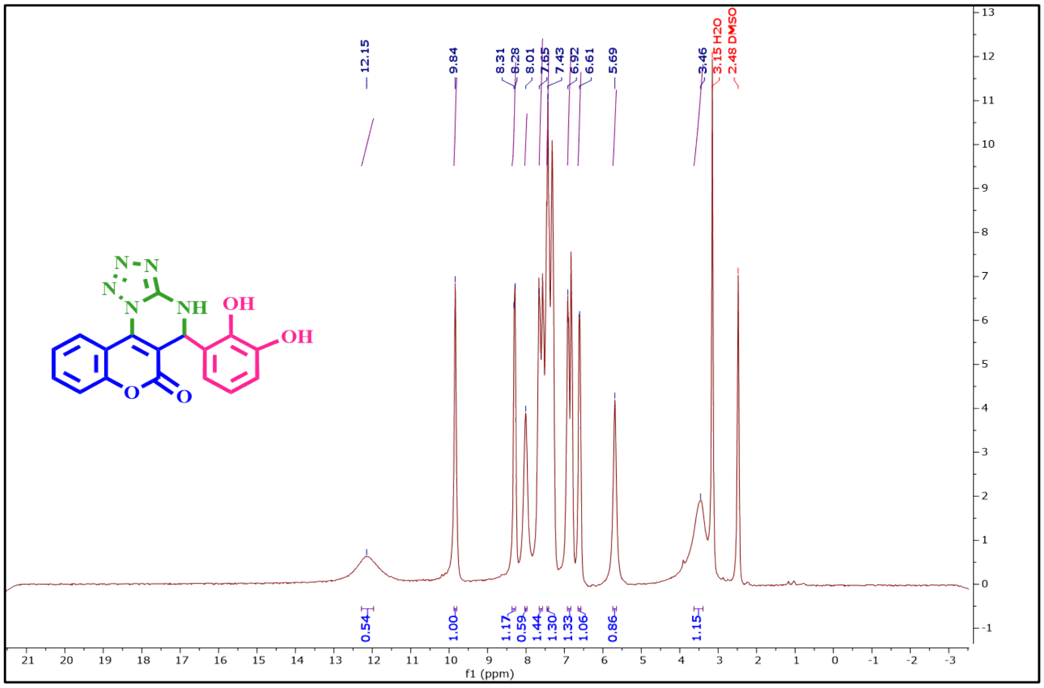

^{1}HNMR Spectrum of 5a

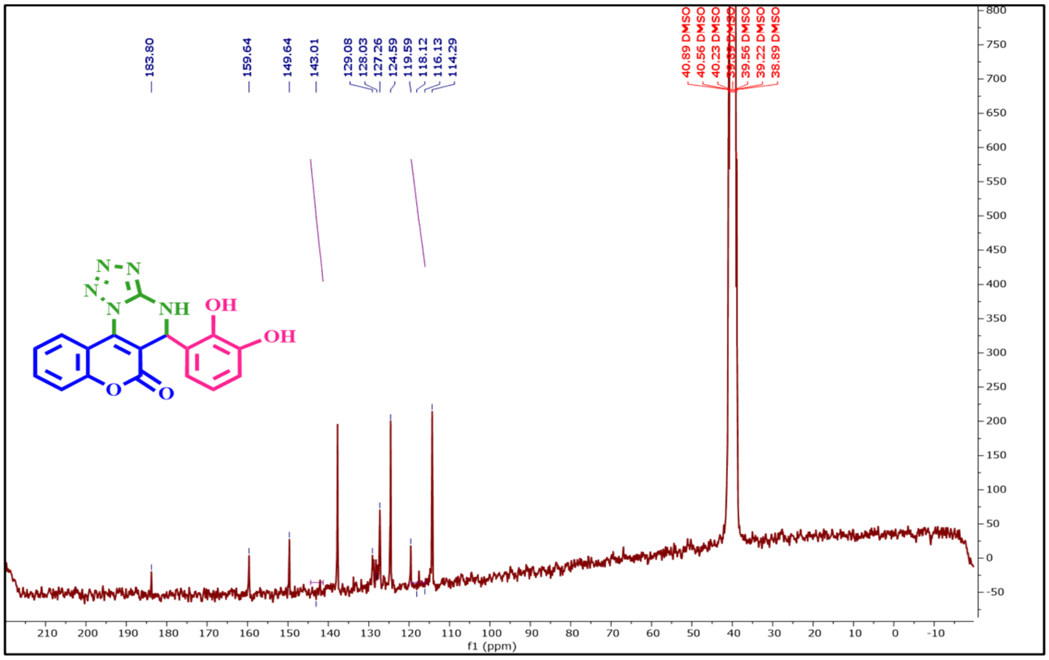

^{13}CNMR Spectrum of 5a

5-(2,4-dichlorophenyl)-4,5-dihydro-6*H*-chromeno[3,4-*e*] tetrazolo[1,5-*a*] pyrimidin-6-one **(6a)**

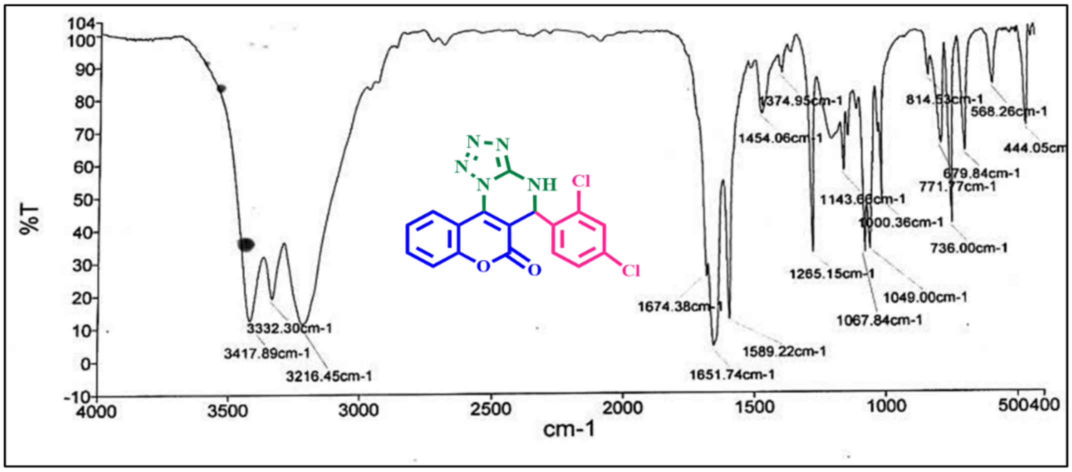

IR Spectrum of 6a

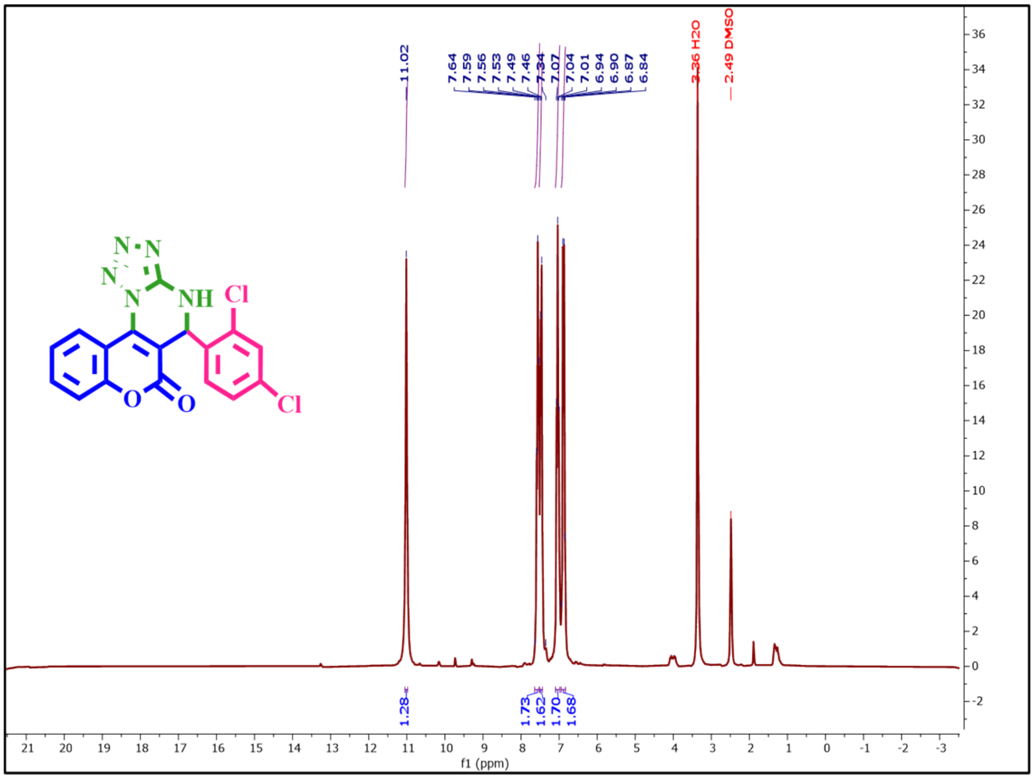

^{1}HNMR Spectrum of 6a

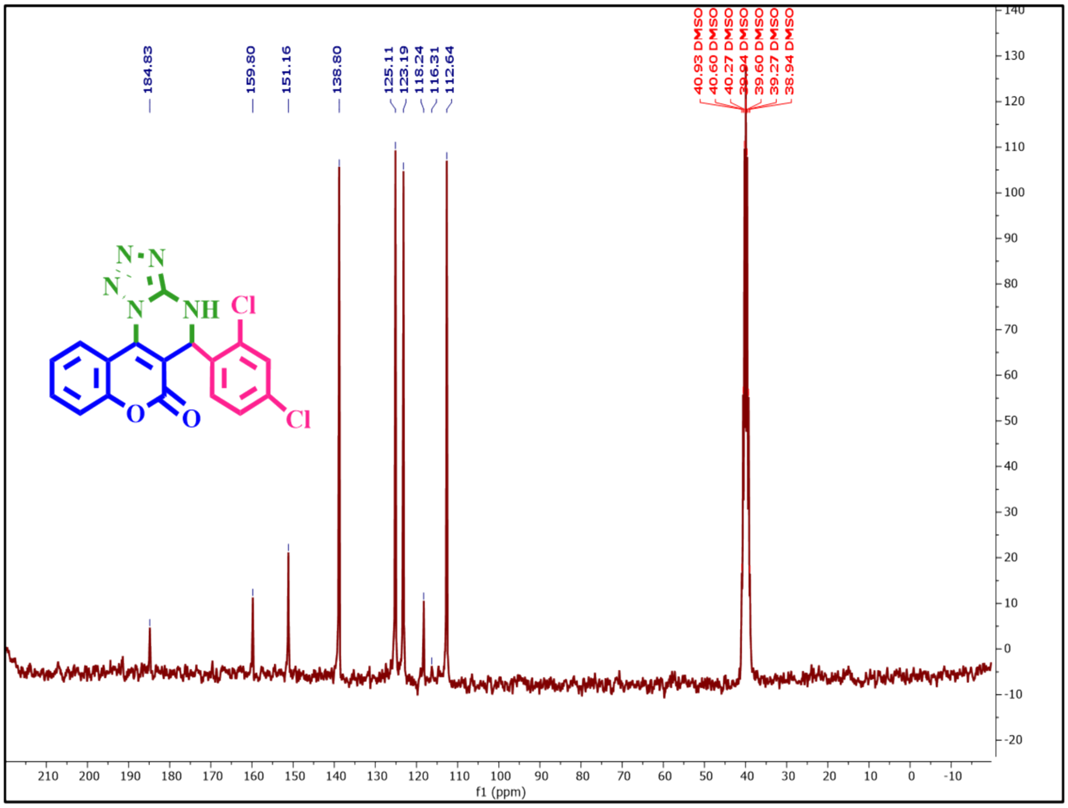

^{13}CNMR Spectrum of 6a

5-(2-methoxyphenyl)-5,9-dihydropyrimido[5,4-*e*]tetrazolo[1,5-*a*]pyrimidine-6,8(4*H*,7*H*)-dione **(7a)**

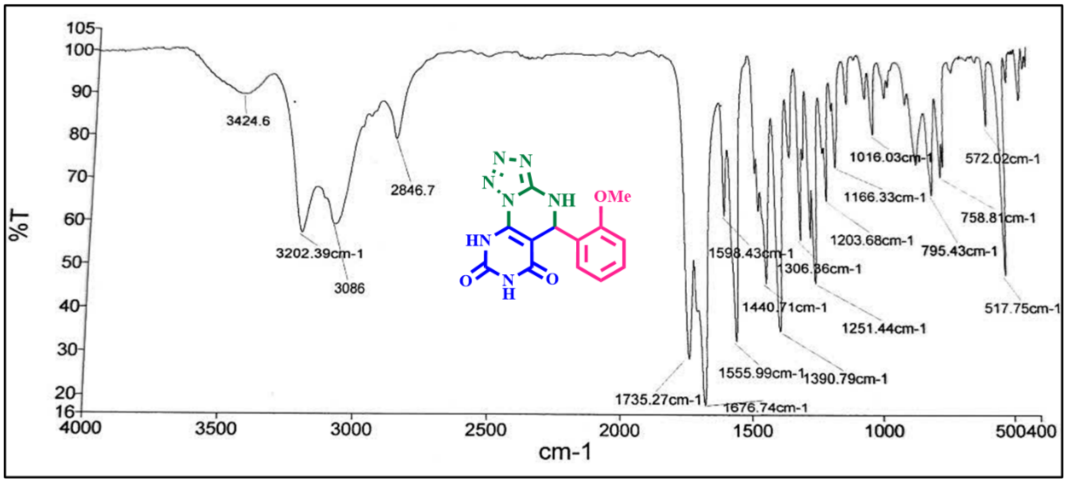

IR Spectrum of 7a

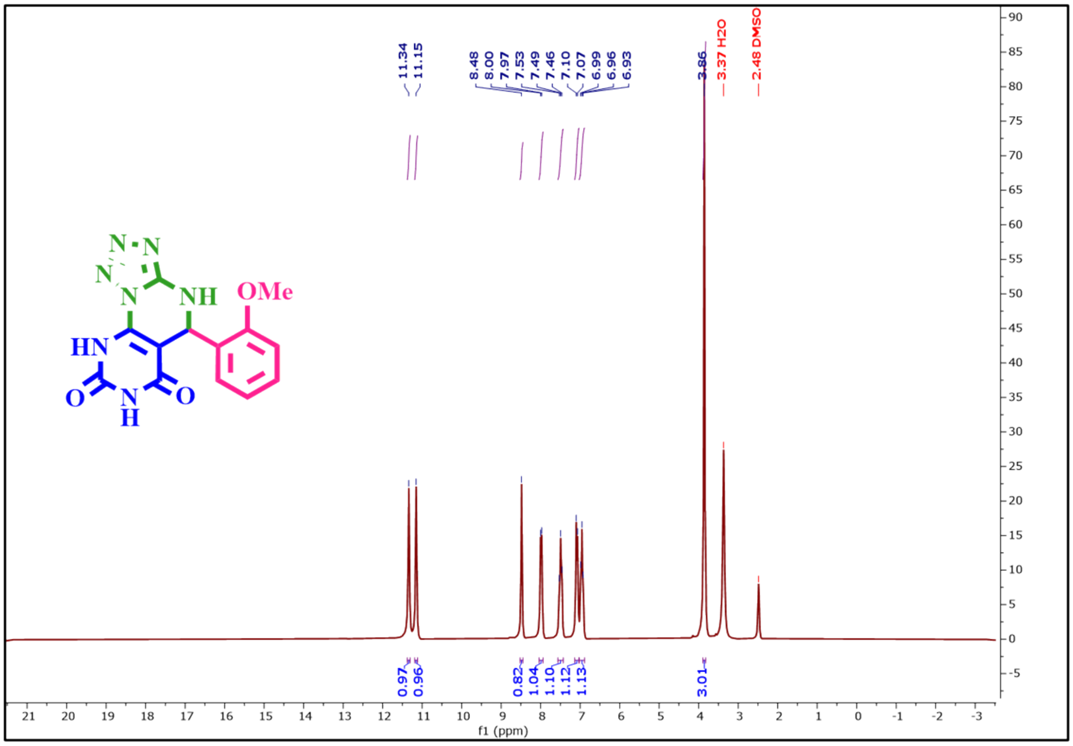

^{1}HNMR Spectrum of 7a

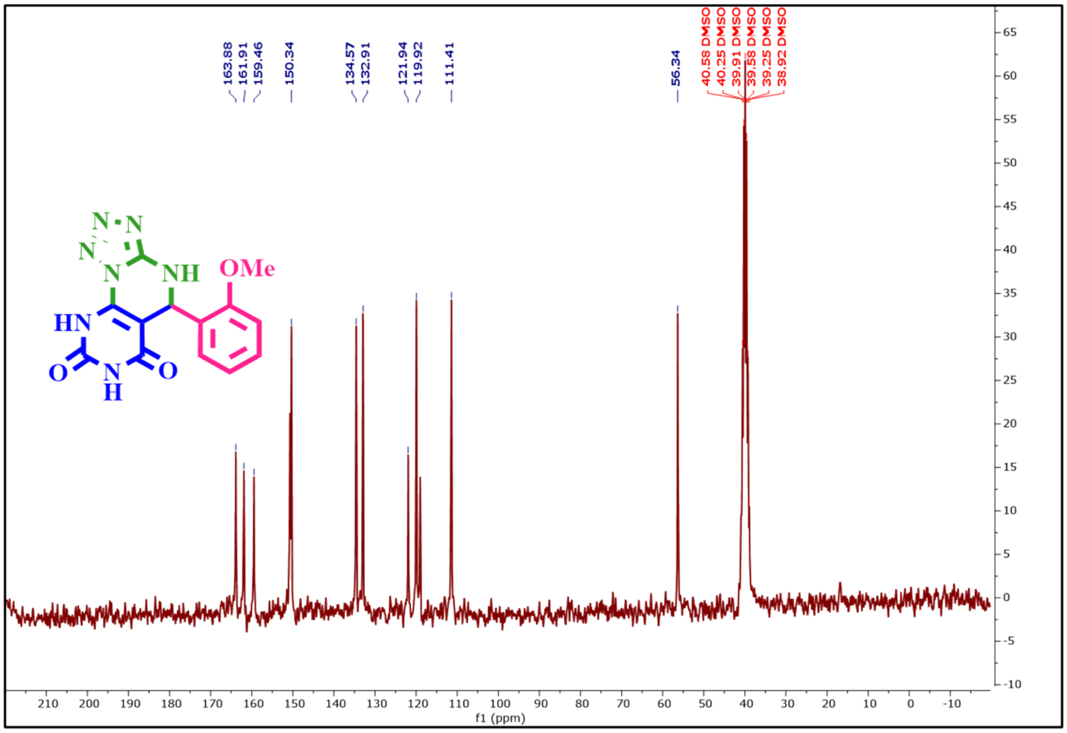

^{13}CNMR Spectrum of 7a

5-(thiophen-2-yl)-5,9-dihydropyrimido[5,4-*e*] tetrazolo[1,5-*a*] pyrimidine-6,8(4*H*,7*H*)-dione **(8a)**

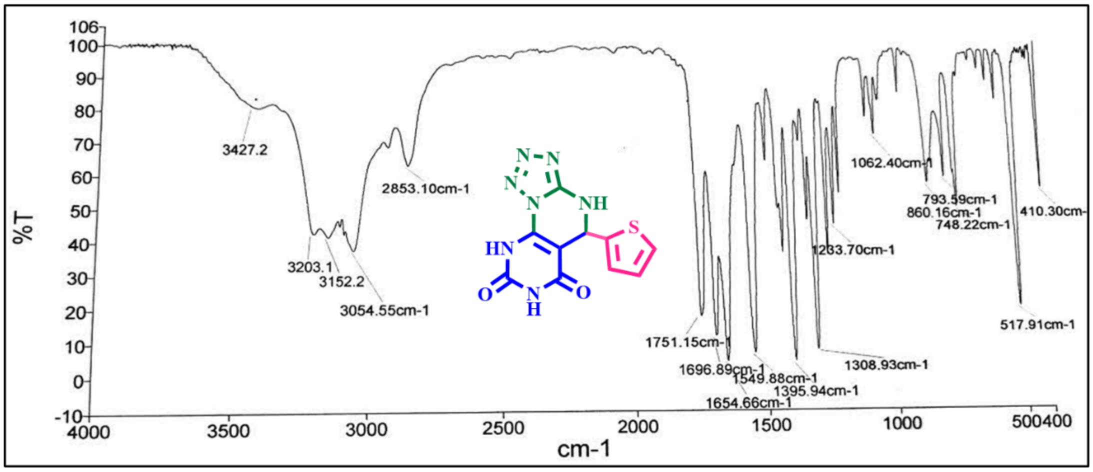

IR Spectrum of 8a

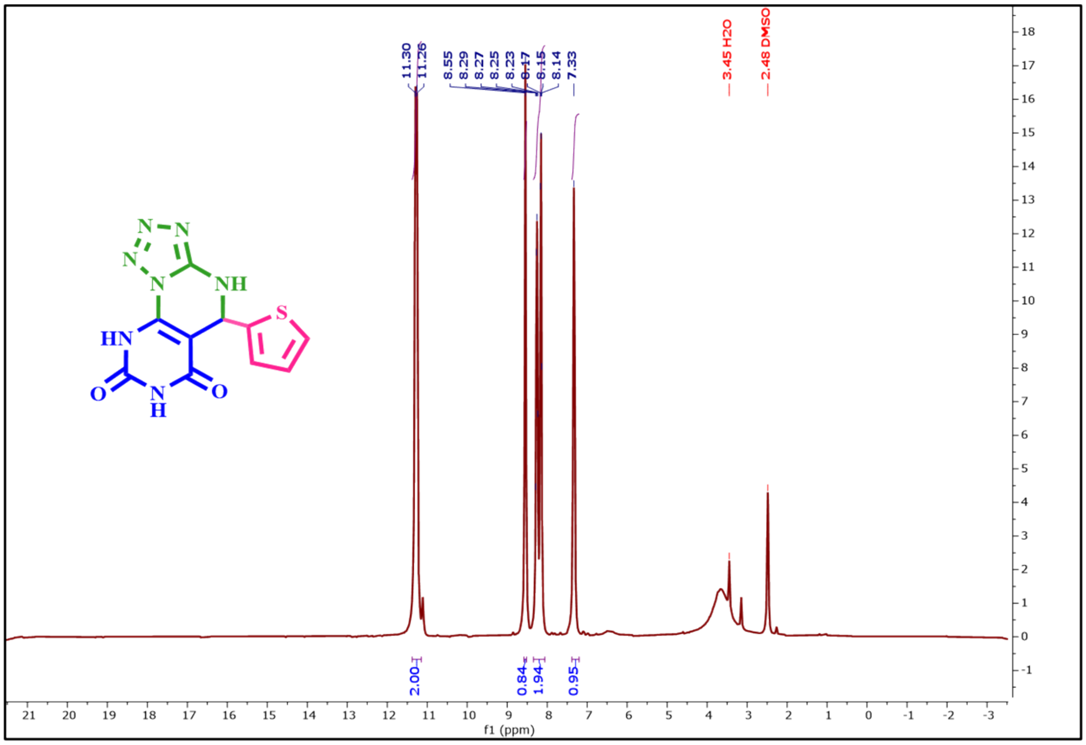

^{1}HNMR Spectrum of 8a

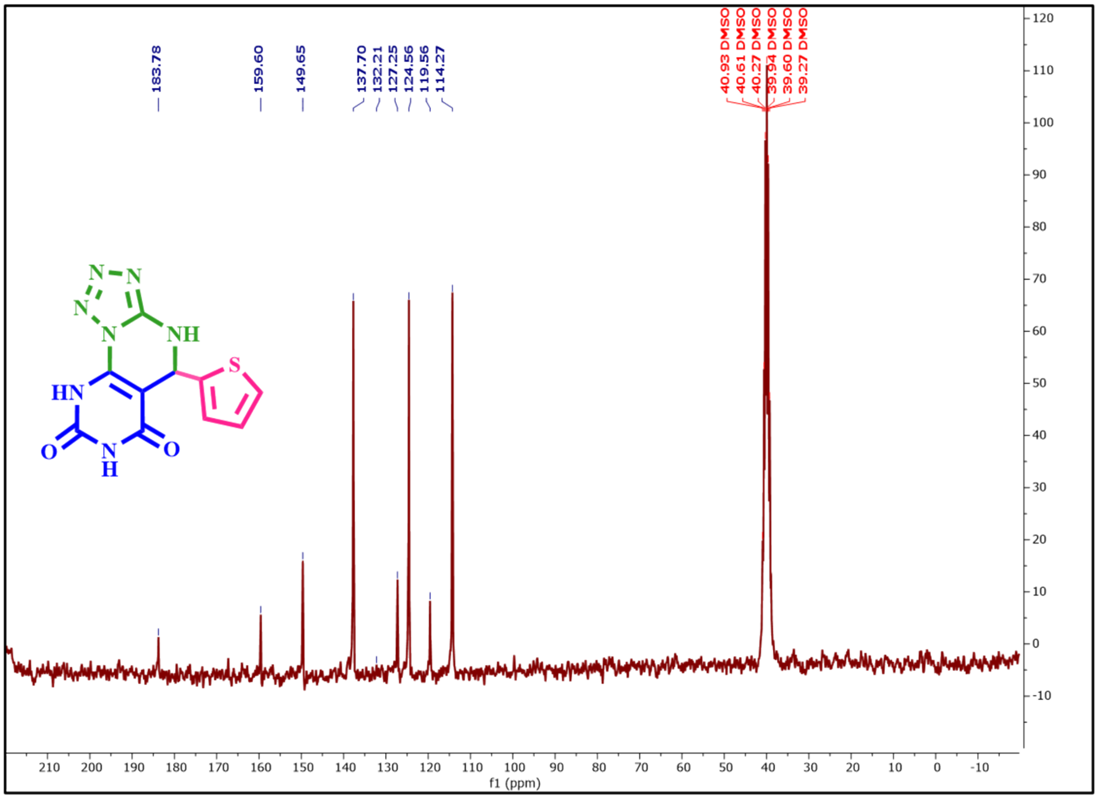

^{13}CNMR Spectrum of 8a

8,8-dimethyl-5-phenyl-5,7,8,9-tetrahydrotetrazolo[1,5-*a*]quinazolin-6(4*H*)-one **(9a)**

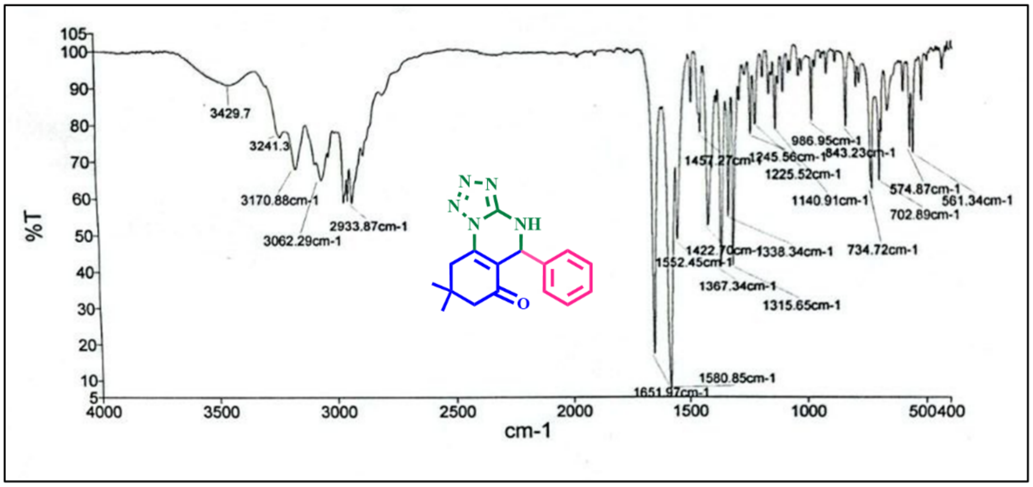

IR Spectrum of 9a

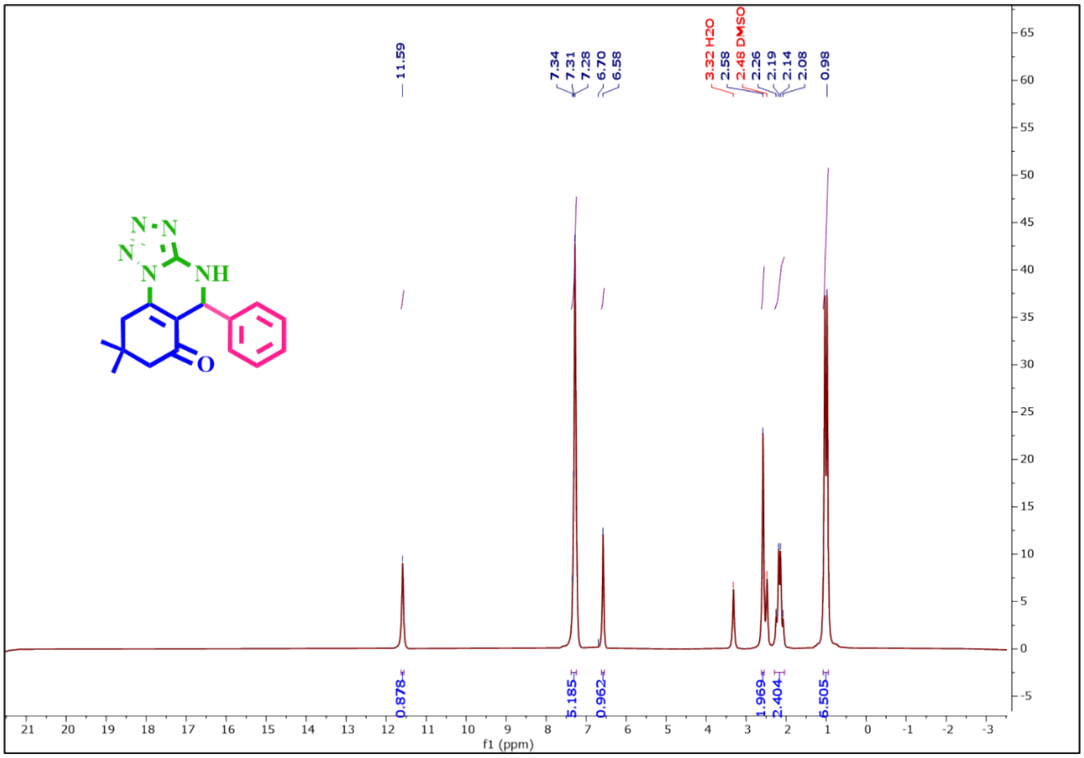

^{1}HNMR Spectrum of 9a

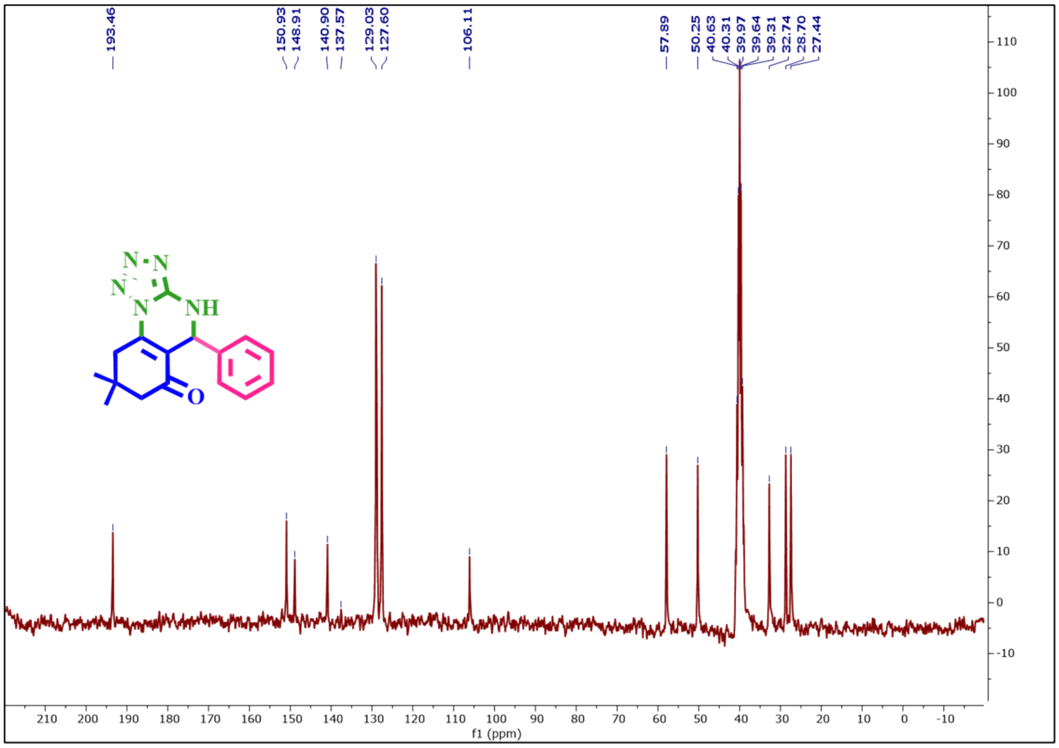

^{13}CNMR Spectrum of 9a

5-(4-chlorophenyl)-8,8-dimethyl-5,7,8,9-tetrahydrotetrazolo[1,5-*a*]quinazolin-6(4*H*)-one

(10a)

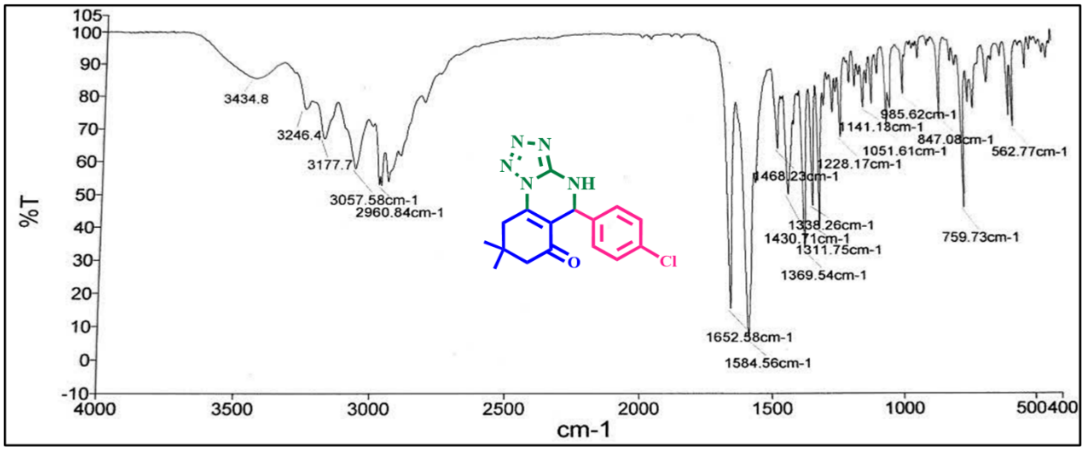

IR Spectrum of 10a

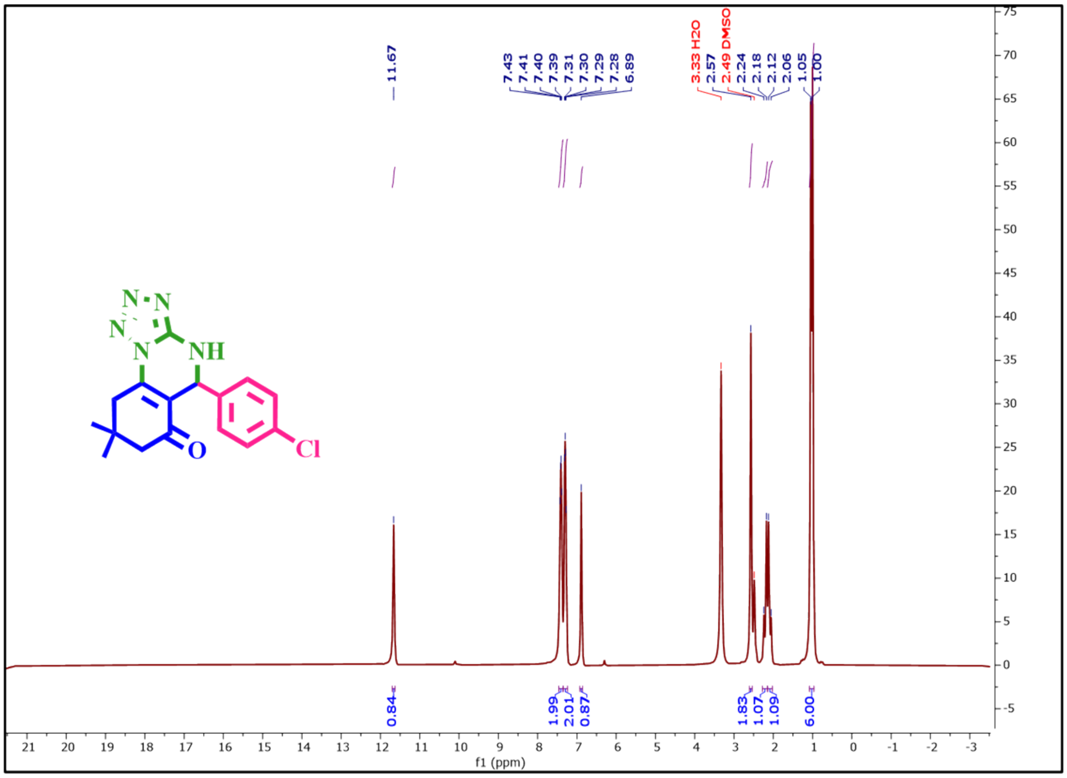

^{1}HNMR Spectrum of 10a

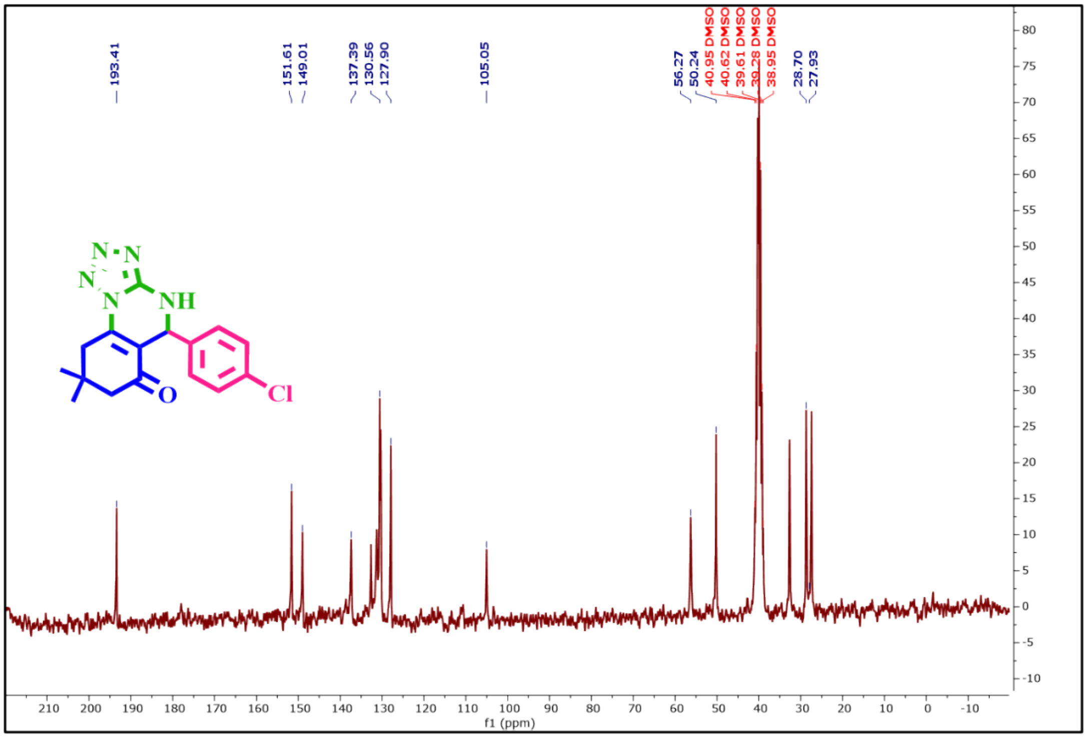

^{13}CNMR Spectrum of 10a

5-(3-ethoxy-4-hydroxyphenyl)-8,8-dimethyl-5,7,8,9-tetrahydrotetrazolo[1,5-*a*] quinazolin - 6(4*H*)-one **(11a)**

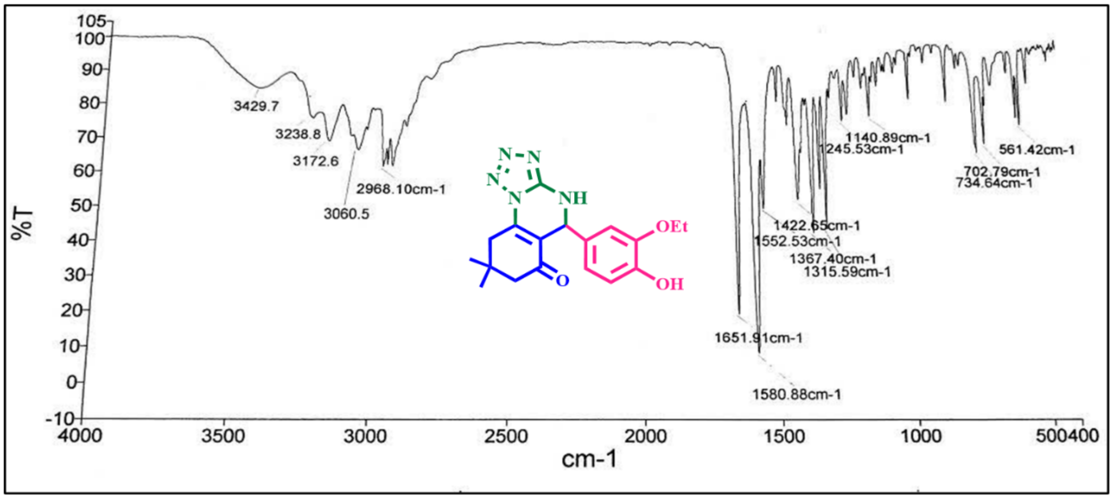

IR Spectrum of 11a

5-(2-chlorophenyl)-8,8-dimethyl-5,7,8,9-tetrahydrotetrazolo[1,5-*a*] quinazolin-6(4*H*)-one **(12a)**

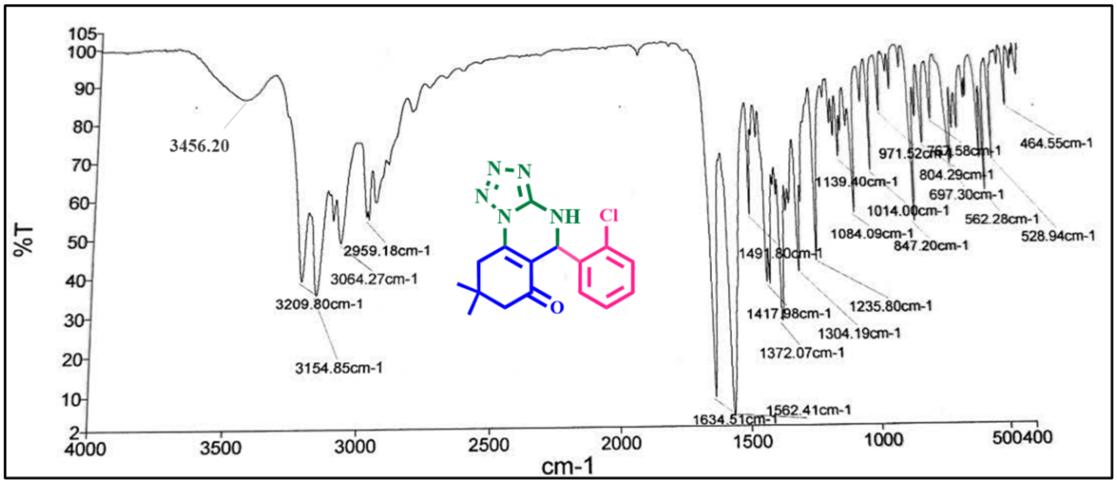

IR Spectrum of 12a
